# Estimating the contribution of key populations towards HIV transmission in South Africa

**DOI:** 10.1002/jia2.25650

**Published:** 2021-02-02

**Authors:** Jack Stone, Christinah Mukandavire, Marie‐Claude Boily, Hannah Fraser, Sharmistha Mishra, Sheree Schwartz, Amrita Rao, Katharine J Looker, Matthew Quaife, Fern Terris‐Prestholt, Alexander Marr, Tim Lane, Jenny Coetzee, Glenda Gray, Kennedy Otwombe, Minja Milovanovic, Harry Hausler, Katherine Young, Mfezi Mcingana, Manezi Ncedani, Adrian Puren, Gillian Hunt, Zamakayise Kose, Nancy Phaswana‐Mafuya, Stefan Baral, Peter Vickerman

**Affiliations:** ^1^ Population Health Sciences University of Bristol Bristol United Kingdom; ^2^ Department of Infectious Disease Epidemiology London School of Hygiene and Tropical Medicine London United Kingdom; ^3^ Department of Infectious Disease Epidemiology Imperial College London United Kingdom; ^4^ St Michaels Hospital University of Toronto Toronto Canada; ^5^ Department of Epidemiology Johns Hopkins Bloomberg School of Public Health Baltimore MD USA; ^6^ London School of Hygiene and Tropical Medicine London United Kingdom; ^7^ University of California San Francisco San Francisco CA USA; ^8^ Equal International Washington DC USA; ^9^ Perinatal HIV Research Unit Faculty of Health Sciences University of the Witwatersrand Johannesburg South Africa; ^10^ South African Medical Research Council Cape Town South Africa; ^11^ TB HIV Care Cape Town South Africa; ^12^ National Institute of Communicable Diseases Johannesburg South Africa; ^13^ Research and Innovation Office North West University Potchefstroom South Africa

**Keywords:** mathematical modelling, population attributable fraction, key populations, female sex workers, clients, men who have sex with men

## Abstract

**Introduction:**

In generalized epidemic settings, there is insufficient understanding of how the unmet HIV prevention and treatment needs of key populations (KPs), such as female sex workers (FSWs) and men who have sex with men (MSM), contribute to HIV transmission. In such settings, it is typically assumed that HIV transmission is driven by the general population. We estimated the contribution of commercial sex, sex between men, and other heterosexual partnerships to HIV transmission in South Africa (SA).

**Methods:**

We developed the “Key‐Pop Model”; a dynamic transmission model of HIV among FSWs, their clients, MSM, and the broader population in SA. The model was parameterized and calibrated using demographic, behavioural and epidemiological data from national household surveys and KP surveys. We estimated the contribution of commercial sex, sex between men and sex among heterosexual partnerships of different sub‐groups to HIV transmission over 2010 to 2019. We also estimated the efficiency (HIV infections averted per person‐year of intervention) and prevented fraction (% IA) over 10‐years from scaling‐up ART (to 81% coverage) in different sub‐populations from 2020.

**Results:**

Sex between FSWs and their paying clients, and between clients with their non‐paying partners contributed 6.9% (95% credibility interval 4.5% to 9.3%) and 41.9% (35.1% to 53.2%) of new HIV infections in SA over 2010 to 2019 respectively. Sex between low‐risk groups contributed 59.7% (47.6% to 68.5%), sex between men contributed 5.3% (2.3% to 14.1%) and sex between MSM and their female partners contributed 3.7% (1.6% to 9.8%). Going forward, the largest population‐level impact on HIV transmission can be achieved from scaling up ART to clients of FSWs (% IA = 18.2% (14.0% to 24.4%) or low‐risk individuals (% IA = 20.6% (14.7 to 27.5) over 2020 to 2030), with ART scale‐up among KPs being most efficient.

**Conclusions:**

Clients of FSWs play a fundamental role in HIV transmission in SA. Addressing the HIV prevention and treatment needs of KPs in generalized HIV epidemics is central to a comprehensive HIV response.

## 1 INTRODUCTION

Despite high HIV prevalence’s among key populations (KPs) such as female sex workers (FSW, 39.5% to 71.8% [1, 2, 3, 4, 5, 6, 7, 8, 9, 10, 11]) and men who have sex with men (MSM, 13.2% to 58.4% [11, 12, 13, 14, 15, 16, 17, 18, 19, 20, 21, 22, 23, 24, 25, 26, 27]) in South Africa (SA), the HIV response has traditionally focussed on the general population, based on the assumption that KPs play a small role in HIV transmission in generalized HIV epidemics. This assertion has largely been based on the UNAIDS modes of transmission (MOT) model, a static model which represents risk in a single year, and generally estimates that <30% of annual HIV infections in sub‐Saharan Africa (SSA) are acquired by KPs (FSW, MSM, clients of FSW and people who inject drugs (PWID)) [28]. Although the Incidence Patterns Model (IPM) was developed to address limitations of the MOT model [29], important limitations remain. Specifically, both models only make short‐term projections of HIV acquisition and so do not account for onward HIV transmissions resulting from infections among KPs. Also, the IPM does not include partners of KPs. These limitations have been highlighted by other dynamic transmission modelling analyses that have shown that commercial sex [30, 31, 32, 33, 34, 35], non‐commercial sex of clients [30], or sex between men [30, 35, 36] can be important contributors to HIV transmission in SSA. However, all but two (Kenya and SA [32, 33]) analyses focussed on West Africa, with only the Kenya analysis [32] suggesting that commercial sex is important elsewhere in Africa. Neither analysis considered MSM.

Although SA has nearly achieved the UNAIDS HIV treatment targets, the rate of new HIV infections is over twice UNAIDS targets [37]. To determine whether further impact could be achieved through added initiatives among KPs, it is important to understand how their unmet treatment and prevention needs are contributing to HIV transmission. Our analysis estimates the long‐term contribution of commercial sex, sex between men and other heterosexual partnerships to HIV transmission in SA, and evaluates the potential impact of increased antiretroviral therapy (ART) coverage among different sub‐populations.

## 2 METHODS

### 2.1 Model description

**Figure 1:**
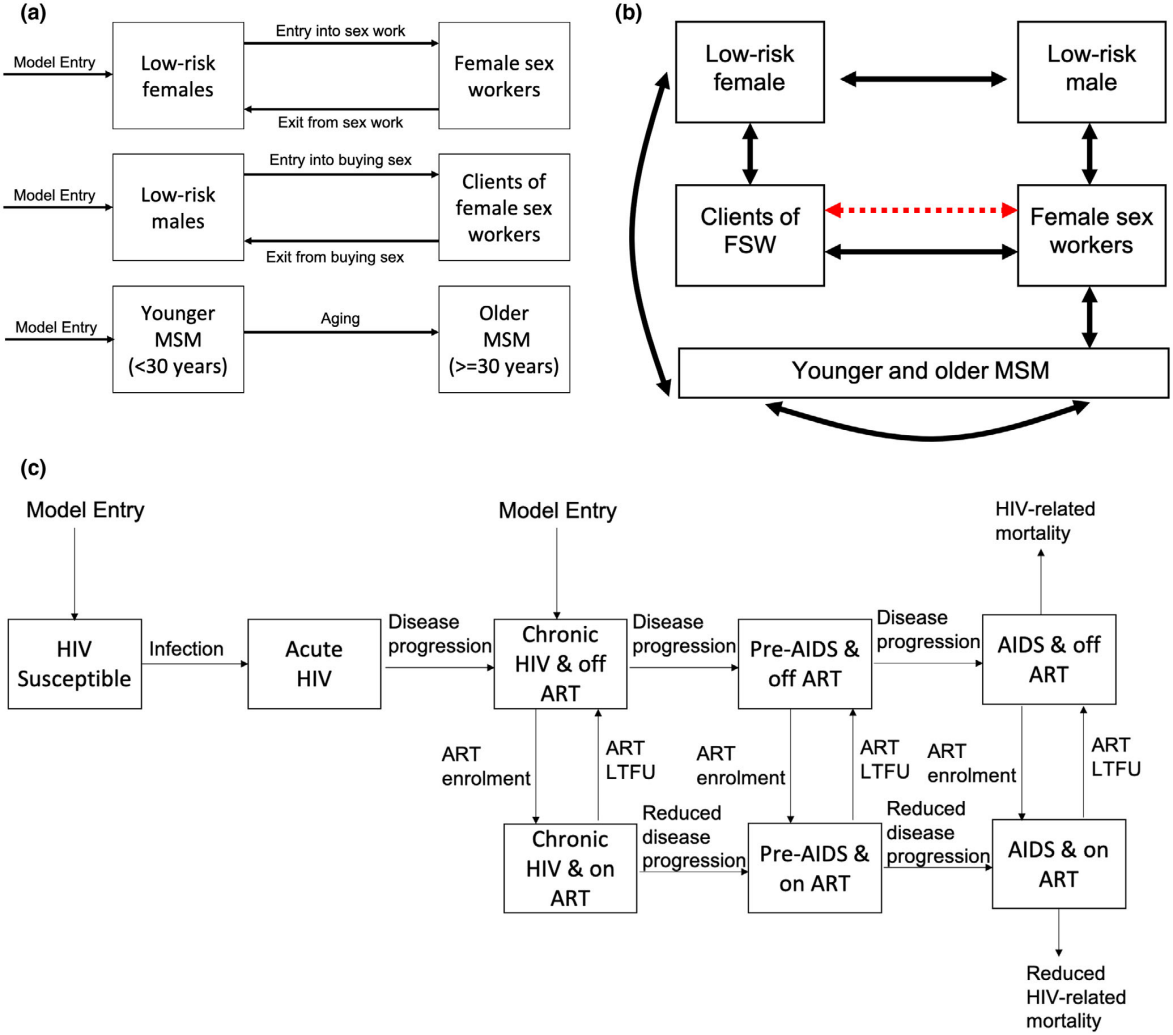
Model schematics illustrating the (**a**) movement of individuals in and out of different sub‐populations (**b**) sexual interactions which can result in HIV transmission among low‐risk females, low‐risk males, female sex workers, their clients and men who have sex with men and (**c**) stratification of the population with respect to HIV infection. Red dashed arrow in (**b**) denotes commercial sex and all other arrows denote sex with main and casual partners. Note that non‐related HIV mortality not shown in (**c**) for clarity. LTFU denotes loss to ART care; FSW denotes female sex workers; MSM denotes men who have sex with men.

We adapted a dynamic model of HIV transmission ([35]; “Key‐Pop model”) among adults (15 to 49 years) that divides the population into low‐risk females and males, FSW, clients of FSW, young MSM (<30 years) and older MSM (≥30 years; Figure 1). MSM are stratified by age because most MSM surveys only capture young MSM. Low‐risk individuals are defined as people that are not MSM and currently do not engage in commercial sex. The model did not include PWID because they make‐up only 0.2% of SA’s adult population [38] and have a low HIV prevalence compared to other KP (21%) [39].

Individuals enter the modelled population as low‐risk males, low‐risk females or young MSM when they become sexually active, at rates that balance non‐HIV deaths (all sub‐populations) and ageing out of the model (at age 50) and incorporating population growth. Low‐risk males and females can become clients and FSWs respectively, at rates that balance cessation from these groups, which results in them returning to the low‐risk groups. MSM age from the younger to older group.

The model captures HIV transmission through vaginal and anal intercourse between all males and females, and anal intercourse among MSM (Figure 1b). The model incorporates HIV infection and disease progression (Figure 1c). Upon infection, susceptible individuals acquire acute HIV infection, then progress to chronic infection, pre‐AIDS and AIDS. Chronically infected individuals or those with more advanced disease can initiate ART, which reduces HIV‐related disease progression and AIDS‐related mortality. Individuals on ART can be lost to care.

HIV transmission occurs due to main, casual and commercial sexual partnerships. Commercial partnerships only occur between FSWs and their clients. Main and casual partnerships between men only occur among MSM, also including paid sex between men. Non‐commercial heterosexual main and casual partnerships occur between all groups (Figure 1c). An individual’s risk of HIV acquisition is related to the HIV prevalence of their sexual partners, and the frequency and type of sex acts within different partnerships. HIV transmission is elevated during acute infection and pre‐AIDS and reduced if on ART, with individuals with AIDS not engaging in sex. Condom use also reduces transmission risk, which varies by partnership type and is time‐dependent. An increasing proportion of males are circumcised over time, which reduces their risk of HIV acquisition during insertive sex. Sexual partnerships are balanced by allowing the sexual behaviour of males with females to determine female sexual behaviour. The model excluded the recent introduction of PrEP in SA due to the low coverage levels achieved (<5% among HIV‐negative MSM/FSW). The model is described Appendix S1.

### 2.2 Model parameterization, calibration and validation

Population size estimates for SA and death rates for 1985 to 2020 were obtained from the United Nations Population Division [40]. FSW size estimates for 2013 came from a national study [41] using multiple methodologies [42]. Reliable estimates of client population size do not exist, and so were estimated indirectly through balancing the number of commercial sex partners reported by FSWs and clients [43]. A range for the number of MSM was derived based on estimates (and 95% confidence intervals) of the proportion of men who report same‐sex behaviour from various SA surveys [12, 44, 45, 46] (see Supplementary Materials).

Data for FSWs came from recent FSWs surveys (2013 to 2016) in multiple cities [3, 4, 5, 6, 47, 48], with older studies being used to determine behavioural and prevalence trends over time. One HIV‐incidence estimate was available from Durban (1996 to 2000) [49]. Unpublished client data came from two surveys in Port Elizabeth (2017/2018) and Klerksdorp (2018) [50].

Data for MSM came from 22 cross‐sectional surveys [11, 12, 13, 14, 15, 16, 17, 18, 19, 20, 21, 22, 23, 24, 25, 26, 27, 45, 46, 51, 52, 53, 54, 55, 56, 57, 58, 59, 60] undertaken over 1988 to 2017 in multiple cities and nationally [54, 58]. HIV incidence estimates came from three studies [59, 61, 62]. Low‐risk adult sexual behaviour data came from five national surveys undertaken over 2002 to 2017 [44, 63, 64, 65, 66]. Details of these studies are in Table S1, with a summary of parameter and calibration data in Table 1, and full details in the Tables S2‐S5.

**Table 1:** Summary of main prior parameter ranges and calibration data (most recent estimates) used for different population groups

	Low risk males	Low risk females	FSW	Clients	Young MSM	Older MSM
Calibration data						
Size estimate (% of adult male or female population)	Depends on % Clients	99.04% to 99.31% (2013)	0.69% to 0.96% (2013)	Balance commercial sex acts	0.65% to 7.3% (2002 to 2008)	
HIV prevalence (2014 to 2019)	13.3% to 16.5%^a^	24.3% to 28.2%^b^	49.2% to 61.5%	14.0% to 28.7%	28.4% to 54.8%	
ART coverage	50.0% to 60.0%^a^ (2018)	60.0% to 70.0%^b^ (2018)	39.1% to 59.3% (2014 to 2015)	26.7% to 37.3% (2017 to 2018)	11.4% to 27.8% (2015 to 2016)	
Model parameters % with partners						
Main heterosexual	75.0% to 83.0%	71.0% to 77.0%	25.0% to 90.0%	81.0% to 98.0%	2.8% to 40.6%	
Casual heterosexual	8.0% to 49.0%	2.0% to 27.0%	5.6% to 29.0%	53.4% to 97.9%	6.5% to 17.0%	OR^d^: 1.02 to 2.42
Main MSM	–	–	–	–	46.0% to 77.5%	
Casual MSM	–	–	–	–	60.1% to 74.4%	
Frequency of partners per year^c^						
Commercial sex	–	–	100 to 1000	2.3 to 72.8	–	–
Main heterosexual	1.1 to 2.8	1.0 to 1.3	1.0 to 3.07	1.0 to 2.9	1.0 to 2.68	IRR^d^: 0.44 to 0.94
Casual heterosexual	1.7 to 2.9	1.7 to 2.9	1.0 to 18.0	1.1 to 15.1	1.0 to 6.5	IRR^d^: 1.06 to 2.21
Main MSM	–	–	–	–	1.0 to 2.01	IRR^d^: 1.09 to 1.54
Casual MSM	–	–	–	–	1.0 to 4.0	IRR^d^: 1.12 to 1.59
% of commercial sex acts that are anal	–	–	0.6% to 9.3%^e^		–	–
Frequency of vaginal sex acts among heterosexual partners						
Main partners (per year)	42.0 to 70.8	42.0 to 70.8	24 to 144	6 to 144	12 to 120	
Casual partners (per partner)	1.1 to 4.3	1.1 to 4.3	0.2 to 8.3	0.5 to 10.2	2.0 to 6.5	
Frequency of anal sex acts among heterosexual partners						
Main partners (per year)	1.2 to 13.2	1.2 to 13.2	1.6 to 60.0	0 to 7.2	0 to 28	
Casual partners (per partner)	0.02 to 0.26	0.0 to 0.22	0.0 to 3.3	0.0 to 0.52	0.0 to 5.0	
Frequency of anal sex for men with men per year						
Main partners (per year)	–	–	–	–	12 to 120	
Casual partners (per partner)	–	–	–	–	2.0 to 10.7	

Note this is included to give a summary with full details of the data used to calibrate the model and prior parameter ranges in the Supplementary Materials. FSW, female sex workers; MSM, men who have sex with men.

^a^ Among all males

^b^ among all females

^c^ among those with partners

^d^ compared to younger MSM

^e^ the rest are vaginal.

**Figure 2:**
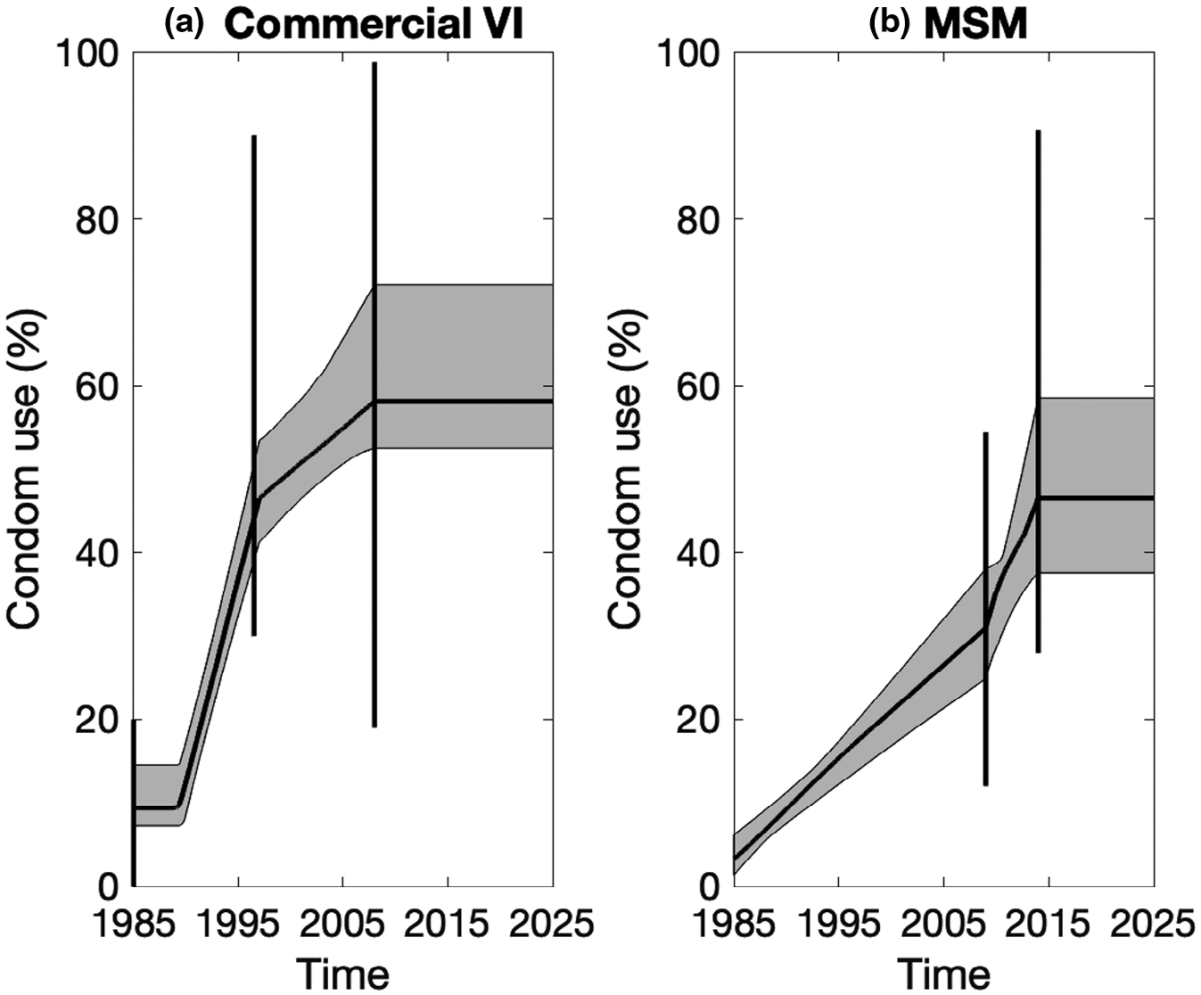
Estimated condom use trends for (**a**) commercial sex (vaginal intercourse ‐ VI) for female sex workers (FSW) and (**b**) male main and casual partners of men who have sex with men (MSM). For commercial sex, we assume condom use for anal intercourse is 0.5 to 1 times that of vaginal intercourse for all years. Continuous black line indicates median projections from all the baseline model fits with shaded areas showing 95% CrI. Vertical black lines show prior ranges.

Based on FSW survey data [1, 2, 3, 4, 44, 47, 50, 67], condom use during last commercial vaginal intercourse was assumed to increase over time as in Figure 2. The wide ranges account for inherent uncertainties in self‐reported measures of condom use and differences in reporting by FSW and clients [1, 2, 3, 4, 6, 44, 47, 50, 67]. Based on data from Port Elizabeth, we assumed a relative risk of 0.5 to 1 for condom use during commercial anal intercourse compared to commercial vaginal intercourse. Based on MSM survey data [21, 22, 23, 24, 25, 26, 58], condom use in last anal intercourse for all male partnerships of MSM was assumed to increase as in Figure 2, with the same levels of condom use assumed for their female partners [26].

Time trends in adult ART coverage for SA came from UNAIDS [68]. ART coverage among people living with HIV increased from 0% in 2003 to 50.0% to 60.0% and 60.0% to 70.0% for adult male and females, respectively, by 2018 (Figure S1). For FSWs, ART coverage was assumed to be 0.7 to 1 times that of adult females based on FSW self‐reported ART data for 2013 to 2016 [2, 3, 47, 48]. For clients, data from 2017 to 2018 also suggested low ART coverage (32.3% to 37.3%; self‐reported) and low viral suppression (26.7% to 29.0%; tested), so we assumed their ART coverage was 0.7 to 1 times that of adult males. For MSM, self‐reported data suggested lower ART coverage (9.7% to 28% for 2012 to 2016 [24, 26, 69, 70]) than other groups and low viral suppression (22% to 23% in 2015 [26]), so we assumed their ART coverage was 0.3 to 0.75 times that of adult males. Figure S2 shows trends in ART coverage. ART initiation rates were calibrated to data on ART coverage while assuming yearly attrition rates of 6.4% to 18.2% among all groups [17, 71, 72], except for FSWs where we assumed a higher yearly attrition rate (22.7% to 38.6% [5]). All other biological parameters came from the literature (Table S5).

Uncertainty ranges were assigned to all model parameters, with all parameters remaining constant over time except those already described and the in flow of HIV‐infected 15‐year‐olds, which varied based on available data. The model was calibrated using an approximate Bayesian computation sequential Monte Carlo (ABC SMC) method, [73] which accounts for uncertainty in the calibration data and parameters and ranks model runs by their goodness of fit. The ABC method was used to calibrate the model to estimates of the population size, ART coverage, HIV incidence among adults, and HIV prevalence among all males and females, FSWs and MSM. The ABC SMC begins with 10,000 parameter sets sampled from prior distributions using Latin Hypercube sampling, which are then successively perturbed to improve their goodness of fit. The ABC routine produced a set of 10,000 baseline model fits which were used to give the median and 95% credibility intervals (95% CrI; 2.5th to 97.5th percentile range) for all model projections. Appendix S1 includes further details.

HIV epidemiological data not used for model calibration (two prevalence estimates for clients, one incidence estimate for FSW and three for MSM) were used to cross‐validate the model fits.

### 2.3 Model analyses

#### 2.3.1 Transmission population attributable fraction

The contribution of sex within or between specific subgroups (e.g. commercial sex between FSW and clients) to the overall new HIV infections over a specific time‐period (tPAF (transmission population attributable fraction)) was estimated over 10‐years for 2010 to 2019 and 2020 to 2029, and over 30‐years for 1990 to 2019. The tPAF was estimated as the percentage of new HIV infections prevented over the time‐period if the transmission risk due to a specific partnership type was set to zero over that time‐period.

#### 2.3.2 Impact of existing interventions

We estimated the HIV‐impact of historical increases in ART coverage, male circumcision and condom use up to 2019. The historical impact was estimated by comparing projections of baseline model fits with counter‐factual scenarios where there was either no condom use after 1985, no scale‐up in male circumcision after 2002, or no‐one started ART from 2003 (when coverage of these interventions were negligible).

#### 2.3.3 Implications for scaling‐up interventions

To assess the implications of our tPAF findings to intervention planning, we estimated the proportion of new HIV infections prevented over 10‐years from 2020 (prevented fraction), of scaling‐up ART coverage to 81% among each population group separately; equivalent to UNAIDS targets of 90% of HIV‐infected individuals being diagnosed and 90% of those being on ART. We also evaluated their efficiency through estimating the number of infections averted per additional person‐year of ART among that risk group over 2020 to 2030.

#### 2.3.4 Uncertainty analyses

We undertook an analysis of covariance (ANCOVA) across the baseline model fits to determine which parameter uncertainties contribute most to the variability in the 10‐year tPAF (2010 to 2019) for those partnership types with the largest tPAF.

Ethical approval and consent were not required as no new data were collected for this study. The data used in this study were collected between 1996 and 2018; see Table S1.

## 3 RESULTS

### 3.1 Baseline model projections

**Figure 3:**
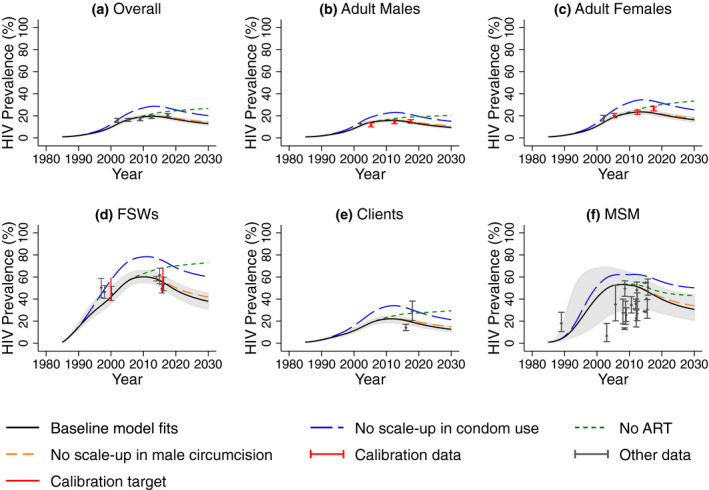
A comparison of median and 95% credibility intervals from baseline model fits (black line and shaded area) with HIV prevalence estimates for (**a**) overall adult population, (**b**) adult male and (**c**) adult female general population, (**d**) female sex workers (FSWs), (**e**) their clients, and (**f**) men who have sex with men (MSM) and HIV prevalence projections without ART (green line), without scale‐up in male circumcision (orange line) and without condom use (blue line). Red points and whiskers show data with 95% confidence intervals used for model calibration, and black points show cross validation data not used in model calibration but shown to compare with model projections.

**Figure 4:**
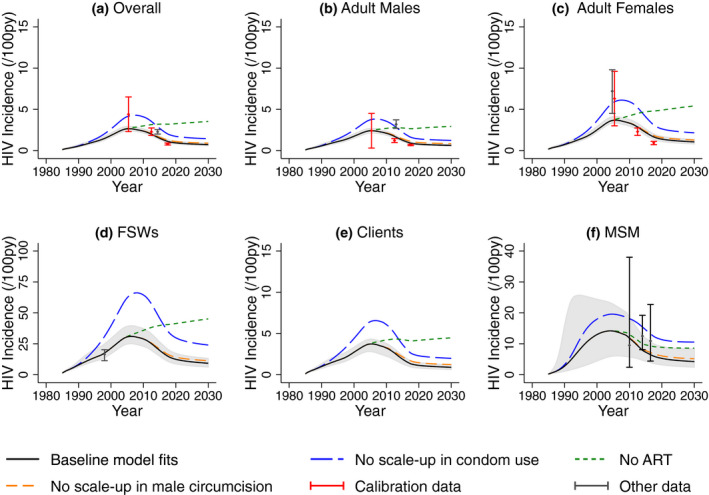
A comparison of median and 95% credibility intervals from baseline model fits (black line and shaded area) with HIV incidence estimates for (**a**) overall adult population, (**b**) adult male and (**c**) adult female general population, (**d**) female sex workers (FSWs), (**e**) their clients, and (**f**) men who have sex with men (MSM) and HIV incidence projections without ART (green line), without scale‐up in male circumcision (orange line) and without condom use (blue line). Black points show cross validation data not used in model calibration but shown to validate the model projections. Note that the scale for the y‐axes are different for panels (d) and (f).

Figures 3 and 4 show the baseline model agrees well with the calibration data and cross validation data on HIV prevalence and incidence not used for model calibration (see Appendix S1 and Figure S3). Model projections suggest that HIV prevalence in all risk groups peaked approximately 2010, has decreased since then, and is expected to stabilize around 2030 if interventions stay at current levels. Similar trends are seen for HIV incidence (Figure 4), although incidence peaked approximately 2005, has decreased more, and will stabilize earlier. Projections of HIV deaths are in Figure S4.

### 3.2 Contribution of different partnership types to overall HIV transmission

**Figure 5:**
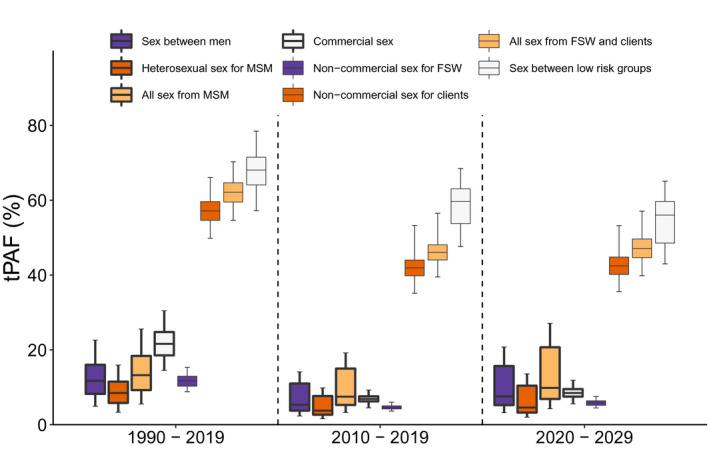
Ten and thirty‐year transmission population attributable fraction (tPAF) for different sexual partnership types. The tPAF is estimated as the proportion of all HIV infections prevented if the HIV transmission risk due to a specific type of sexual behaviour is removed over the following time periods: 1990 to 2019, 2010 to 2019 and 2020 to 2029. The box plots signify the uncertainty (middle line is median, limits of boxes are the 25% and 75% percentiles, and whiskers are 2.5% and 97.5% percentile range) in the tPAF estimates due to uncertainty in the model parameters. FSW, female sex workers; MSM, men who have sex with men.

Model projections (Figure 5) suggest that over 2010 to 2019, the risk behaviour contributing most to ongoing HIV transmission was sex between low‐risk males and females, which if fully protected (i.e. all transmission and acquisition risk removed) could prevent 59.7% (95% CrI: 47.6% to 68.5%) of new HIV infections over this period (tPAF). Next was non‐commercial sex of clients (with low‐risk females or FSWs), which contributed 41.9% (35.1% to 53.2%) of new HIV infections. Otherwise, transmission due to commercial sex contributed 6.9% (4.5% to 9.3%) and sex between MSM contributed 5.3% (2.3% to 14.1%). Similar tPAFs are projected for 2020 to 2029 (Figure 5). Importantly, commercial sex contributed more to HIV transmission (21.6%; 95% CrI: 14.5% to 30.5%) over 1990 to 2019 than it is now.

The analysis of covariance (Figure S5‐S8) showed that variability in the tPAF for non‐commercial sex of clients was mainly due to uncertainty in the HIV prevalence among low‐risk males (41.8% of variation; negative correlation) and clients (24.8% of variation; positive correlation in Figure S5) and the proportion of men that are clients (28.2%, positive correlation in Figure S5). Variability in the tPAF for sex between low‐risk individuals was due to uncertainty in the same parameters and the proportion of men that are MSM (16.5%; negative correlation).

### 3.3 Impact of existing interventions

Model projections (Figure 3) suggest that without ART since 2003, HIV prevalence in 2019 would have been 39.6% (34.9% to 45.9%) higher overall, with 44.9% (40.8% to 51.6%) or 3.3 million (2.9 to 3.9 million) more infections over 2003 to 2019. Similarly, if condom use had not increased since early 1980s, HIV prevalence in 2019 would have been 47.8% (39.4% to 61.2%) higher overall, with 41.2% (33.4% to 52.3%) or 4.7 million (3.9 to 5.9 million) more infections over 1985 to 2019. Lastly, if male circumcision had not increased since 2002, HIV prevalence in 2019 would have been 3.9% (3.5% to 5.8%) higher overall, with 2.7% (2.4% to 4.6%) or 0.2 million (0.2 to 0.4 million) more infections over 2002 to 2019.

### 3.4 Impact of scaling‐up interventions

**Figure 6:**
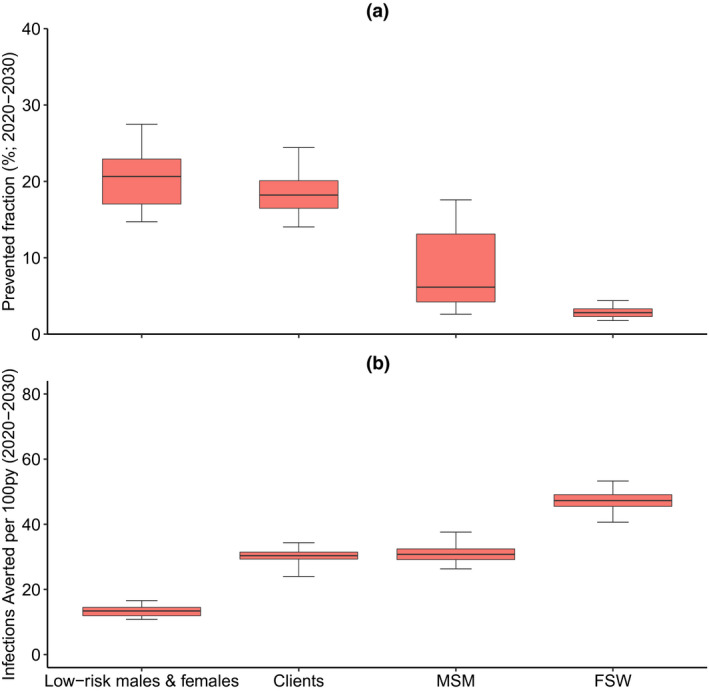
The prevented fraction and efficiency of scaling‐up ART coverage to 81% (UNAIDS 90:90:90 target) within each sub‐group from 2020 to 2030. (**a**) shows the impact of targeting treatment to each sub‐group measured as the prevented fraction (% of new HIV infections that could be averted) over 2020 to 2030. (**b**) Shows the efficiency of targeting treatment to each sub‐group, measured as the number of infections averted per 100 py of intervention. The box plots signify the uncertainty (middle line is median, limits of boxes are the 25% and 75% percentiles, and whiskers are 2.5% and 97.5% percentile range) in the impact projections due to uncertainty in the baseline model fits. FSW, female sex workers; MSM, men who have sex with men.

Model projections (Figure 6) suggest that scaling‐up ART among clients or low‐risk individuals would avert most infections, while scaling‐up ART among clients, FSWs and MSM would be most efficient. For instance, scaling‐up ART (to 81% coverage) among low‐risk individuals would avert 20.6% (14.7% to 27.5%) of new HIV infections over 2020 to 2030, with 13.4 (10.8 to 16.6) infections averted per 100 py of ART. In contrast, scaling‐up ART among clients would avert 18.2% (14.0% to 24.4%) of new HIV infections, but with 30.4 (24.0 to 34.3) infections averted per 100 py of ART; 2.3 (1.5 to 2.9) times more than from targeting low‐risk individuals. Although averting fewer infections, scaling‐up ART among MSM and FSW are similarly or more efficient than targeting clients.

## 4 DISCUSSION

Our results suggest that the unmet HIV prevention and treatment needs of clients of FSW contribute substantially to ongoing HIV transmission in SA. Currently, their key risk behaviour for HIV transmission is sex with their non‐commercial sex partners, which if fully protected could prevent nearly half of all new HIV infections in SA over the next 10 years. Although commercial sex was important, contributing one‐fifth of infections since 1990, this has declined due to heightened condom use and the increasing role of other groups as HIV prevalence increased. The large role of clients stems from their large population, paired with their high HIV prevalence (13% to 22%), frequent casual sex and sub‐optimal ART coverage. Principally, our indirect size estimations suggest that 22% (95% CrI 18% to 29%) of adult men buy sex; which is considerable but consistent with other analyses [33].

Existing interventions in SA have had considerable impact, with increases in condom use having prevented five million infections since 1985 and ART having prevented three million since 2003. Going forward, our results clearly indicate where future prevention and treatment activities should be focussed. Although impact will be achieved from scaling‐up ART among low‐risk individuals, as currently undertaken in SA, similar impact and greater efficiency will be achieved from increasing ART among FSWs and their clients, with greatest efficiency being achieved among FSWs. Similar specification should apply for prevention interventions such as HIV pre‐exposure prophylaxis and condom promotion to reduce the susceptibility of FSWs and clients. To date, there is limited investment in specific HIV initiatives for clients in SA [74] or elsewhere across SSA, with prevention responses for commercial sex focussing on FSWs. Clients also remain an understudied group, highlighting the need for further research to better understand their unmet needs in HIV transmission and to inform future HIV prevention and treatment strategies.

## 5 STRENGTHS AND LIMITATIONS

Strengths of our analysis include undertaking a dynamic assessment of the contribution of the unmet prevention and treatment needs of KPs to HIV transmission in a generalized epidemic setting. The calibration of our model to detailed data in a Bayesian framework also increases the rigour of our analyses, with its cross‐validation to incidence and prevalence data and projections of HIV deaths [75, 76, 77] adding extra plausibility to our projections.

The modelling was limited by uncertainty in some model parameters, most crucially the client population size where there is no reliable estimate. Our indirect method estimated the number of clients through balancing the amount of commercial sex reported by FSW and clients, with data from Port Elizabeth suggesting FSWs have 10 to 49 times more commercial sex than clients, so implying a similar greater number of clients. This method relies on an accurate estimate for the number of FSWs, for which we used national estimates based on the Wisdom of the Crowds method [41], validated using a range of other methodologies [78]. Although our indirect method estimates a much larger client population (22%, 95% CrI: 18% to 29% of adult men) than suggested by the HSRC household studies in SA (<2% [44, 63]) and elsewhere in SSA (range: 0.025% to 22.9% [79, 80, 81]), it is well known that these surveys seriously underestimate such behaviours due to social desirability biases [82, 83]. Indeed, other methods, such as anonymous polling booth surveys from Benin and Nigeria suggest ~20% of men have recently bought sex (last month/year) [82, 84]. This agrees with a recent household survey undertaken in a peri‐urban settlement of Johannesburg (Diesploot) using audio computer‐assisted self‐interview, which found that one‐fifth of adult men had sex with a FSW in the last year [85]. These estimates are comparable to our model projections as well as other data on the prevalence of transactional sex (18% to 46%) among men in SA [85, 86]. Although this highlights the need for better studies to estimate this crucial parameter, it also suggests that our estimates of the client population size are in line with estimates using ‘less biased’ methods, lending credence to our results.

The under‐reporting of commercial sex by men in the HSRC studies also meant that we could not distinguish between men who engage in commercial sex and those that do not. HSRC data for all males was therefore used to parameterize the risk behaviours of low‐risk males. This may have resulted in us over‐estimating the sexual behaviour of the low‐risk population and so their contribution to the HIV epidemic.

The modelling was further limited by our reliance on client data from two small SA surveys. To account for uncertainty in this data and its representativeness, parameter estimates incorporated the range across the 95% confidence intervals or inter‐quartile ranges of estimates from each survey, with additional uncertainty being associated with parameters where only one survey provided data. Despite these issues, the use of these surveys should also be considered a strength because no previous modelling analyses for SA have utilized client data. Further studies are needed among clients in SA to update our model projections.

Additionally, studies used diverse measures for risk behaviours, especially condom use, resulting in difficulties in assessing how behaviours changed over time. This was accentuated by likely biases in the reporting of condom use, resulting in uncertainty in the trends used in the model fitting. This uncertainty was constrained through the model calibration and did not affect our model projections. Most ART coverage estimates for KPs were based on self‐report, which may be upwardly biased as demonstrated by three of four studies that also collected viral suppression data. Although further viral suppression data would allow better quantification of ART coverage and its impact, our uncertainty analyses suggest it should not affect our tPAF estimates. Lastly, our model did not stratify by factors affecting risk (violence or stigma); this may have masked the importance of these heterogeneities.

Furthermore, our model focussed only on those of reproductive age (15 to 49). Less than 10% of new infections are estimated to occur in this group [68], and so it is unlikely that this omission would affect our findings. The model also did not incorporate injecting drug use due to PWID making up only 0.2% of the South African adult population [38] and them having a similar HIV prevalence to other sub‐groups (21%) [39]. Although their exclusion is unlikely to have affected our PAF estimates, it is important that future analyses consider their evolving contribution to the wider HIV epidemic and the potential importance of targeting interventions to PWID.”

Finally, we did not consider the resources needed to scale‐up ART in different key populations. Although the calculated efficiency of ART for different sub‐populations gives an idea of their relative cost‐effectiveness, the cost and feasibility of achieving such targets should also be considered when prioritising interventions. Indeed, the costs of providing testing and ART to different population groups is likely to vary due to differences in demand and structural barriers to care. This is demonstrated in previous analyses which find a wide range in the costs of providing HIV testing, treatment and PrEP across different key populations [87, 88], with the costs for key‐populations sometimes being greater than for the general population [89]. We also did not assess the impact and efficiency of prevention interventions, but rather just considered the scale‐up of ART to illustrate how tPAF analyses can be useful for guiding targeting strategies.

## 6 COMPARISON TO OTHER STUDIES

Many HIV epidemic models have been developed for SA, primarily focussing on assessing the impact and cost‐effectiveness of prevention and treatment interventions [90, 91, 92, 93, 94]. However, few have focussed on sex work [33] or other KPs [95]. Although there are numerous dynamic model assessments of the contribution of KPs to HIV epidemics in different African settings [30, 31, 32, 33, 34, 35, 36], only two have considered generalized epidemic settings [32, 33]; one being SA [33]. Our projections of the contribution of commercial sex to HIV transmission are similar to the existing SA analysis (20% to 25% from start of the epidemic to 2010 and 7% in 2010 [33]), but are much lower than was estimated for Kenya (46% after 10 years [32]). The Kenyan study produced higher estimates due to assuming a greater proportion of females engaging in sex work (2.2% vs. 0.83% in our analysis) and using a different measure to estimate the contribution of commercial sex (comparing HIV incidence after 10 years with or without commercial sex). Our modelling extends these existing analyses by also considering the contribution of other sexual risk behaviours and groups, crucially MSM and non‐commercial partnerships of clients, and as a result showing the importance of incorporating KPs into the HIV response, similar to findings from Cote d’Ivoire [30].

## 7 CONCLUSIONS AND IMPLICATIONS

Our analyses for SA add to data from West Africa [30] suggesting that clients of FSW contribute substantially to HIV transmission. Considerable investment has been made in SA to scaling‐up interventions for preventing and treating HIV; including national HIV testing (10 million tested annually) and male circumcision campaigns (0.6 million circumcised annually), and the largest ART programme in the world (3.7 million people currently receive ART [74]); Ours and other analyses suggest these interventions have had large impact [90, 94]. However, although some civil society organizations have provided condoms and HIV testing and counselling to clients alongside their FSW activities, there have been few interventions developed specifically for clients in South Africa and SSA, with most targeting truck drivers, mine workers or military personnel [96, 97]. While recommendations for client interventions in SA have been developed [96], the latest national HIV strategy does not mention clients [74] among new initiatives for key populations [74], including FSW [98] and MSM [99]. Although scaling‐up ART among FSW will be highly efficient, our findings suggest this will not substantially reduce the contribution of clients to HIV transmission and will achieve much less impact than directly targeting clients. Furthermore, general population interventions may reach some clients, but they are unlikely to meet their specific needs. Client focussed interventions could recruit through sex work venues or referrals from FSW, as used in bio‐behavioural surveys of clients [50]. These interventions would then need to meet their needs, including remedying their sub‐optimal levels of viral suppression (27% to 29%), reducing their frequency of casual sex and increasing levels of condom use among main partners (34% to 37%) [50] (Unpublished Port Elizabeth Survey). If this is achieved, our model projections suggest that this new focus on the prevention and treatment needs of clients could achieve substantial impact.

## COMPETING INTEREST

The authors report no conflicts of interests. HF has received an honorarium from MSD unrelated to this research. KL has received funding from GlaxoSmithKline on work outside the submitted work. PV has received honoraria from Gilead and Abbvie unrelated to this research, and unrestricted research funding from Gilead unrelated to this work.

## AUTHOR CONTRIBUTIONS

PV, MCB, SB, SM and SS conceptualized the study. JS performed the model analyses, which were initiated by CM, with advice from HF. PV supervised the project. PV and JS wrote the first draft of the manuscript. JS and CM reviewed the literature for data sources for the model. SB, SS, AR, JC, GG, KO, MM, MQ, AM, TL, MM, ZK, NPM, AP, HH, KY, MN, GH and FTP collected or contributed data for the modelling. HH and KY supported conceptualization of South Africa‐specific research questions. KL, TL, AM and MQ undertook data analyses for parameterizing and calibrating the model. All authors contributed to data interpretation, writing the manuscript and approved the final version.

## 8 ACKNOWLEDGEMENTS

None declared.

### 8.1 FUNDING

This publication resulted in part from research funded by a supplement to the Johns Hopkins University Center for AIDS Research, an NIH funded programme (P30AI094189) and R01NR016650 with support specifically from the Office of AIDS Research (OAR) and the National Institutes of Nursing Research. The programme also received support from Linkages across the Continuum of HIV Services for Key Populations Affected by HIV project (LINKAGES, Cooperative Agreement AID‐OAA‐A‐14‐00045) and the parent study HIV Prevention 2.0 (HP2): Achieving an AIDS‐Free Generation in Senegal (AID‐OAA‐A‐13‐00089). The supplement, LINKAGES and HP2 received support from the United States Agency for International Development (USAID) and the U.S. President’s Emergency Plan for AIDS Relief (PEPFAR). PV also acknowledges support from the NIHR Health Protection Research Unit in Behavioural Science and Evaluation at University of Bristol. SM is supported by a Canadian Institutes of Health and Ontario HIV Treatment Network Research New Investigator Award. JC acknowledges support received through the University of California San Diego, an NIH funded CFAR award (P30 AI036214), funding from the South African Medical Research Council and the Wellcome Trust. The content is solely the responsibility of the authors and does not necessarily represent the official views of any of the funding agencies.

## Supporting information


**Appendix S1.** Supplementary materials

**Figure S1.** a, Modelled condom use trends for low risk males and females. Continuous black line indicates median projections from all the baseline model fits with pink shaded areas showing 95% credibility intervals. Vertical black lines show the prior ranges. b, Modelled condom use trends for female sex workers (FSW). Both figures show condom use for vaginal intercourse (VI). FSW condom use with casual partners for VI is 1.25 to 1.75 times that for main partners VI; condom use for AI with main/casual partners is assumed to be the same as VI; condom use for AI with commercial partners is assumed to be 0.5 to 1.0 times that of commercial VI. Continuous black line indicates median projections from all the baseline model fits with pink shaded areas showing 95% credibility intervals. Vertical black lines show the prior ranges. c, Modelled condom use trends for men how have sex with men (MSM) with their male and female regular and casual partners. Continuous black line indicates median projections from all the baseline model fits with pink shaded areas showing 95% credibility intervals. Vertical black lines show the prior ranges. d, Modelled condom use trends for Clients with their main and casual partners. Condom use is assumed to be the same for vaginal intercourse and anal intercourse. Continuous black line indicates median projections from all the baseline model fits with pink shaded areas showing 95% credibility intervals. Vertical black lines show the prior ranges.
**Figure S2.** Modelled ART trend for female sex workers (FSW), men who have sex with men (MSM) and low risk females and males. Continuous black line indicates median projections from all the baseline model fits with pink shaded areas showing 95% credibility intervals. Vertical black lines show UNAIDS estimates
**Figure S3.** A comparison of model fits with HIV prevalence estimates for (a) younger men who have sex with men (MSM), and (b) older MSM. Continuous black line shows median projections from all the model fits, with the grey shaded areas showing 95% credibility intervals. Red points and whiskers show regional HIV prevalence data points with 95% confidence intervals. Most available MSM data is heavily weighted towards young MSM (e.g. In South African RDS studies of MSM, the median proportion of MSM who are aged <25 is 70%) and so it is to be expected that older MSM will not fit the data so well
**Figure S4.** Model projections of the number of annual HIV deaths. Continuous black line indicates median projections from all the baseline model fits with pink shaded areas showing 95% credibility intervals
**Figure S5.** Scatter plots showing the association between: (a) client population size in 2020 and the population attributable fraction (PAF) of non‐commercial sex among clients from 2020 to 2029; (b) HIV prevalence among clients in 2020 and the PAF of non‐commercial sex among clients from 2020 to 2029; (c) ART coverage among clients in 2020 and the PAF of non‐commercial sex among clients from 2020 to 2029; (d) client population size in 2020 and the PAF of commercial sex from 2020 to 2029; (e) HIV prevalence among clients in 2020 and the PAF of commercial sex from 2020 to 2029; (f) ART coverage among clients in 2020 and the PAF of commercial sex from 2020 to 2029. Black lines are the least‐squares line
**Figure S6.** ANCOVA results: Contribution of uncertainty in each parameter to the variability in the PAF of non‐commercial sex among clients over 2010 to 2019. Parameters accounting for less than 1% of the uncertainty are not shown. MSM, men who have sex with men
**Figure S7.** ANCOVA results: Contribution of uncertainty in each parameter to the variability in the PAF of commercial sex over 2010 to 2019. Parameters accounting for less than 1% of the uncertainty are not shown. FSW, female sex workers; MSM, men who have sex with men; LRM, low risk males
**Figure S8.** ANCOVA Results: Contribution of uncertainty in each parameter to the variability in the PAF of sex between low‐risk groups over 2010 to 2019. Parameters accounting for less than 1% of the uncertainty are not shown. MSM, men who have sex with men
