# Supplementary material for: Estimating the contribution of key populations towards HIV transmission in South Africa
Source: J Int AIDS Soc. 2021 Feb 2;24(1):e25650. doi: 10.1002/jia2.25650 (PMC7855076; doi:10.1002/jia2.25650)
Supplement: Supplementary file 1 — Appendix S1. Supplementary materials [file JIA2-24-e25650-s001.docx]

**Supplementary Materials to: Estimating the contribution of key populations towards HIV transmission in South Africa**

Jack Stone, Christinah Mukandavire, Marie-Claude Boily, Hannah Fraser, Sharmistha Mishra, Sheree Schwartz, Amrita Rao, Katharine Looker, Matthew Quaife, Fern Terris-Prestholt, Alexander Marr, Tim Lane, Jenny Coetzee, Harry Hausler, Katherine Young, Mfezi Mcingana, Manezi Ncedani, Adrian Puren, Gillian Hunt, Zamakayise Kose, Nancy Phaswana-Mafuya, Stefan Baral, Peter Vickerman

# Model description

We used a previously published dynamic HIV transmission model (Mukandavire, Walker et al. 2018) to evaluate the extent to which FSW, clients of FSW (referred to as clients hereafter) and MSM contribute to the overall HIV epidemic in South Africa. The model considers adults (15-49 years), and divides the population into six sub-populations: low-risk females ($i=1$) and males $(i=2$), clients ($i=3$), FSW ($i=4$), young MSM ($<30 years, i=5$) and older MSM ($\geq30 years, i=6$) (Figure 1 shows the model schematic). Low-risk individuals are defined as people that are not MSM and do not report commercial sex.

Individuals enter the modelled population when they become sexually active, at a rate $\Phi$ that balances non-HIV deaths and reflects population growth, with a proportion $p$ entering into the low-risk female group, $\left( 1-p \right)(1-p_{2})$ entering the low-risk male group and $\left( 1-p \right)p_{2}$ entering the young MSM group. Low-risk females become FSW at a rate $\kappa$ and stay as FSW for duration $1/\gamma$ years. Similarly, clients transition from the low-risk male population at rate $k$. Clients remain as clients for a period of $1/g$ years before returning to the low-risk male group. Conversely, MSM move from the young to the older MSM group at a rate $\zeta,$ where they remain until death or ageing out of the model.

The model captures HIV transmission among the sub-populations through vaginal and anal sexual intercourse (VI and AI, respectively) between all males and females, and AI within the MSM group (Figure 1 in main text). The model stratifies the population with respect to HIV infection and disease progression such that for each sub-population$i$, there is uninfected ($S_{i}$), acute infection ($E_{i}$), chronic infection off ART($I_{i}$) or on ART ($I_{i}^{t}$), Pre-AIDS infection off ART ($P_{i}^{t}$) or on ART ($P_{i}^{t}$), and AIDS off ART ($A_{i})$ or on ART ($A_{i}^{t})$, with $i=1,2...6$. Upon infection, susceptible individuals move to the acute stage of HIV infection before progressing to the chronic, pre-AIDS and AIDS phases of infection at fixed rates ($\eta_{1}$for acute to chronic, $\eta_{2}$for chronic to pre-AIDS and $\eta_{3}$for pre-AIDS to AIDS). In the chronic, pre-AIDS and AIDS stages, individuals are recruited onto ART at a rate $\omega_{i}$ (time dependent and differs by risk group). While on ART, HIV-related mortality is reduced by a factor $\varphi$ compared to what it was in the chronic stage. Individuals on ART can be lost to follow-up at a rate $\sigma_{i}$ (which differs by risk group), after which they can be re-recruited onto ART. Individuals with AIDS experience a HIV-related mortality rate $\delta$ which is lowered by a factor $\varphi$if on ART. All sub-populations also experience non-HIV related death and exit the model with rates $\mu_{i}$, which incorporates both non-HIV related death rates and ageing out of the model.

The model incorporates HIV transmission due to main, casual and commercial sexual partnerships. Commercial partnerships can only occur between FSW and their clients, while main and casual partnerships between men only occur amongst the two MSM groups. All other main and casual partnerships between male and females can occur between individuals from all groups, including MSM. The risk of HIV transmission for a particular individual is related to the HIV prevalence of their sexual partners, with the HIV transmission risk being elevated by a factor $\nu$ if they are in the acute stage of infection, and reduced by a factor $\alpha$ if they are on ART, all compared to the transmission risk for the chronic stage. Transmission risk is also related to the average frequency of sex acts (denoted by ${\Psi_{ijh}}^{k})$ for different types of partnerships and between risk groups $i$ and $j$ (with ${\Psi_{ijh}}^{k}={\Psi_{jih}}^{k}$ to ensure they balance) , where $k$ denotes the type of sexual partner (main, casual or commercial) and $h$ denotes the type of sexual act (VI or AI). HIV transmission is reduced through condom use by a factor $\left( 1-\varepsilon\pi_{ijh}^{k} \right),$ where $\varepsilon$is the efficacy of condom use and $\pi_{ijh}^{k}$ is the average consistency of condom use reported by those in risk groups $i$ and $j$ (with $\pi_{ijh}^{k}=\pi_{jih}^{k}$ to ensure they balance). The consistency of condom use is assumed to be time dependent and varies depending on the type of partnership. The model assumes a proportion $\xi$ of males are circumcised, with the model assuming these males have a reduced risk of HIV acquisition, modelled by a factor (1-$\xi\vartheta$), where $\vartheta$ is the efficacy of circumcision.

# Model equations

Low risk female population

$$\frac{dS_{1}}{dt}=\left( 1-\Phi_{1} \right)p\theta+\gamma S_{4}-{(\Lambda}_{1}^{m}+\Lambda_{1}^{c})S_{1}-(\kappa+\mu_{1})S_{1}$$

$$\frac{dE_{1}}{dt}={(\Lambda}_{1}^{m}+\Lambda_{1}^{c})S_{1}+\gamma E_{4}-(\kappa+\eta_{1}+\mu_{1})E_{1}$$

$$\frac{dI_{1}}{dt}=\Phi_{1}p\theta+\eta_{1}E_{1}+\sigma_{1}I_{1}^{t}+\gamma I_{4}-\left( \kappa+\omega_{1}+\mu_{1}+\eta_{2} \right)I_{1}$$

$$\frac{dP_{1}}{dt}=\eta_{2}I_{1}+\sigma_{1}P_{1}^{t}+\gamma P_{4}-\left( \kappa+\omega_{1}+\mu_{1}+\eta_{3} \right)P_{1}$$

$$\frac{dA_{1}}{dt}=\eta_{3}P_{1}+\sigma_{1}A_{1}^{t}+\gamma A_{4}-\left( \kappa+\omega_{1}+\mu_{1}+\delta\right)A_{1}$$

$$\frac{dI_{1}^{t}}{dt}= \omega_{1}I_{1}+\gamma I_{4}^{t}-\left( \kappa+\sigma_{1}+\varphi\eta_{2}+\mu_{1} \right)I_{1}^{t}$$

$$\frac{dP_{1}^{t}}{dt}=\varphi\eta_{2}I_{1}^{t}+\omega_{1}P_{1}+\gamma P_{4}^{t}-\left( \kappa+\sigma_{1}+\varphi\eta_{3}+\mu_{1} \right)P_{1}^{t}$$

$$\frac{dA_{1}^{t}}{dt}= \varphi\eta_{3}P_{1}^{t}+\omega_{1}A_{1}+\gamma A_{4}^{t}-\left( \kappa+\sigma_{1}+\varphi\delta+\mu_{1} \right)A_{1}^{t}$$

Low risk male population

$$\frac{dS_{2}}{dt}=\left( 1-\Phi_{2} \right)(1-p)(1-p_{2})\theta+gS_{3}-{(\Lambda}_{2}^{m}+\Lambda_{2}^{c})S_{2}-(z+\mu_{2})S_{2}$$

$$\frac{dE_{2}}{dt}={(\Lambda}_{2}^{m}+\Lambda_{2}^{c})S_{2}+gE_{3}-(z+\eta_{1}+\mu_{2})E_{2}$$

$$\frac{dI_{2}}{dt}=\Phi_{2}\left( 1-p \right)(1-p_{2})\theta+\eta_{1}E_{2}+\sigma_{2}I_{2}^{t}+gI_{3}-\left( z+\omega_{2}+\mu_{2}+\eta_{2} \right)I_{2}$$

$$\frac{dP_{2}}{dt}=\eta_{2}I_{2}+\sigma_{2}P_{2}^{t}+gP_{3}-\left( z+\omega_{2}+\mu_{2}+\eta_{3} \right)P_{2}$$

$$\frac{dA_{2}}{dt}=\eta_{3}P_{2}+\sigma_{2}A_{2}^{t}+gA_{3}-\left( z+\omega_{2}+\mu_{2}+\delta\right)A_{2}$$

$$\frac{dI_{2}^{t}}{dt}= \omega_{2}I_{2}+gI_{3}^{t}-\left( z+\sigma_{2}+\varphi\eta_{2}+\mu_{2} \right)I_{2}^{t}$$

$$\frac{dP_{2}^{t}}{dt}=\varphi\eta_{2}I_{2}^{t}+\omega_{2}P_{2}+gP_{3}^{t}-\left( z+\sigma_{2}+\varphi\eta_{3}+\mu_{2} \right)P_{2}^{t}$$

$$\frac{dA_{2}^{t}}{dt}= \varphi\eta_{3}P_{2}^{t}+\omega_{2}A_{2}+gA_{3}^{t}-\left( z+\sigma_{2}+\varphi\delta+\mu_{2} \right)A_{2}^{t}$$

Client population

$$\frac{dS_{3}}{dt}=zS_{2}-{(\Lambda}_{3}^{m}+\Lambda_{3}^{c})S_{3}-(g+\mu_{3})S_{3}$$

$$\frac{dE_{3}}{dt}={(\Lambda}_{3}^{m}+\Lambda_{3}^{c})S_{3}+zE_{2}-(g+\eta_{1}+\mu_{3})E_{3}$$

$$\frac{dI_{3}}{dt}=\eta_{1}E_{3}+\sigma_{3}I_{3}^{t}+zI_{2}-\left( g+\omega_{3}+\mu_{3}+\eta_{2} \right)I_{3}$$

$$\frac{dP_{3}}{dt}=\eta_{2}I_{3}+\sigma_{3}P_{3}^{t}+zP_{2}-\left( g+\omega_{3}+\mu_{3}+\eta_{3} \right)P_{3}$$

$$\frac{dA_{3}}{dt}=\eta_{3}P_{3}+\sigma_{3}A_{3}^{t}+zA_{2}-\left( g+\omega_{3}+\mu_{3}+\delta\right)A_{3}$$

$$\frac{dI_{3}^{t}}{dt}= \omega_{3}I_{3}+zI_{2}^{t}-\left( g+\sigma_{3}+\varphi\eta_{2}+\mu_{3} \right)I_{3}^{t}$$

$$\frac{dP_{3}^{t}}{dt}=\varphi\eta_{2}I_{3}^{t}+\omega_{3}P_{3}+zP_{2}^{t}-\left( g+\sigma_{3}+\varphi\eta_{3}+\mu_{3} \right)P_{3}^{t}$$

$$\frac{dA_{3}^{t}}{dt}= \varphi\eta_{3}P_{3}^{t}+\omega_{3}A_{3}+zA_{2}^{t}-\left( g+\sigma_{3}+\varphi\delta+\mu_{3} \right)A_{3}^{t}$$

FSW population

$$\frac{dS_{4}}{dt}=\kappa S_{1}-{(\Lambda}_{4}^{m}+\Lambda_{4}^{c}+\Lambda_{4}^{co})S_{4}-(\gamma+\mu_{4})S_{4}$$

$$\frac{dE_{4}}{dt}={(\Lambda}_{4}^{m}+\Lambda_{4}^{c}+\Lambda_{4}^{co})S_{4}+\kappa E_{1}-(\gamma+\eta_{1}+\mu_{4})E_{4}$$

$$\frac{dI_{4}}{dt}=\eta_{1}E_{4}+\sigma_{4}I_{4}^{t}+\kappa I_{1}-\left( \gamma+\omega_{4}+\mu_{4}+\eta_{2} \right)I_{4}$$

$$\frac{dP_{4}}{dt}=\eta_{2}I_{4}+\sigma_{4}P_{4}^{t}+\kappa P_{1}-\left( \gamma+\omega_{4}+\mu_{4}+\eta_{3} \right)P_{4}$$

$$\frac{dA_{4}}{dt}=\eta_{3}P_{4}+\sigma_{4}A_{4}^{t}+\kappa A_{1}-\left( \gamma+\omega_{4}+\mu_{4}+\delta\right)A_{4}$$

$$\frac{dI_{4}^{t}}{dt}= \omega_{4}I_{4}+\kappa I_{4}^{t}-\left( \gamma+\sigma_{4}+\varphi\eta_{2}+\mu_{4} \right)I_{4}^{t}$$

$$\frac{dP_{4}^{t}}{dt}=\varphi\eta_{2}I_{4}^{t}+\omega_{4}P_{4}+\kappa P_{1}^{t}-\left( \gamma+\sigma_{4}+\varphi\eta_{3}+\mu_{4} \right)P_{4}^{t}$$

$$\frac{dA_{4}^{t}}{dt}= \varphi\eta_{3}P_{4}^{t}+\omega_{4}A_{4}+\kappa A_{1}^{t}-\left( \gamma+\sigma_{4}+\varphi\delta+\mu_{4} \right)A_{4}^{t}$$

MSM population-young

$$\frac{dS_{5}}{dt}=\left( 1-\Phi_{2} \right)(1-p)p_{2}\theta+gS_{3}-{(\Lambda}_{5}^{m}+\Lambda_{5}^{c}+\Lambda_{5*}^{m}+\Lambda_{5*}^{c})S_{2}-(\zeta+\mu_{5})S_{5}$$

$$\frac{dE_{5}}{dt}={(\Lambda}_{5}^{m}+\Lambda_{5}^{c}+\Lambda_{5*}^{m}+\Lambda_{5*}^{c})S_{5}-(\zeta+\eta_{1}+\mu_{5})E_{5}$$

$$\frac{dI_{5}}{dt}=\Phi_{2}\left( 1-p \right)p_{2}\theta+\eta_{1}E_{5}+\sigma_{5}I_{5}^{t}-\left( \zeta+\omega_{5}+\mu_{5}+\eta_{2} \right)I_{5}$$

$$\frac{dP_{5}}{dt}=\eta_{2}I_{5}+\sigma_{5}P_{5}^{t}-\left( \zeta+\omega_{5}+\mu_{5}+\eta_{3} \right)P_{5}$$

$$\frac{dA_{5}}{dt}=\eta_{3}P_{5}+\sigma_{5}A_{5}^{t}-\left( \zeta+\omega_{5}+\mu_{5}+\delta\right)A_{5}$$

$$\frac{dI_{5}^{t}}{dt}= \omega_{5}I_{5}-\left( \zeta+\sigma_{5}+\varphi\eta_{2}+\mu_{5} \right)I_{5}^{t}$$

$$\frac{dP_{5}^{t}}{dt}=\varphi\eta_{2}I_{5}^{t}+\omega_{5}P_{5}-\left( \zeta+\sigma_{5}+\varphi\eta_{3}+\mu_{5} \right)P_{5}^{t}$$

$$\frac{dA_{5}^{t}}{dt}= \varphi\eta_{3}P_{5}^{t}+\omega_{5}A_{5}-\left( \zeta+\sigma_{5}+\varphi\delta+\mu_{5} \right)A_{5}^{t}$$

MSM population-older

$$\frac{dS_{6}}{dt}=\zeta S_{5}-{(\Lambda}_{6}^{m}+\Lambda_{6}^{c}+\Lambda_{6*}^{m}+\Lambda_{6*}^{c})S_{6}-(\mu_{6})S_{6}$$

$$\frac{dE_{6}}{dt}={(\Lambda}_{6}^{m}+\Lambda_{6}^{c}+\Lambda_{6*}^{m}+\Lambda_{6*}^{c})S_{6}+\zeta E_{5}-(\eta_{1}+\mu_{6})E_{6}$$

$$\frac{dI_{6}}{dt}=\eta_{1}E_{6}+\sigma_{6}I_{6}^{t}+\zeta I_{5}-\left( \omega_{6}+\mu_{6}+\eta_{2} \right)I_{6}$$

$$\frac{dP_{6}}{dt}=\eta_{2}I_{6}+\sigma_{6}P_{6}^{t}+\zeta P_{5}-\left( \omega_{6}+\mu_{6}+\eta_{3} \right)P_{6}$$

$$\frac{dA_{6}}{dt}=\eta_{3}P_{6}+\sigma_{6}A_{6}^{t}+\zeta A_{5}-\left( \omega_{6}+\mu_{6}+\delta\right)A_{6}$$

$$\frac{dI_{6}^{t}}{dt}= \omega_{6}I_{6}+\zeta I_{5}^{t}-\left( \sigma_{2}+\varphi\eta_{2}+\mu_{6} \right)I_{6}^{t}$$

$$\frac{dP_{6}^{t}}{dt}=\varphi\eta_{2}I_{6}^{t}+\omega_{6}P_{6}+\zeta P_{5}^{t}-\left( \sigma_{6}+\varphi\eta_{3}+\mu_{6} \right)P_{6}^{t}$$

$$\frac{dA_{6}^{t}}{dt}= \varphi\eta_{3}P_{6}^{t}+\omega_{6}A_{6}+\zeta A_{5}^{t}-\left( \sigma_{6}+\varphi\delta+\mu_{6} \right)A_{6}^{t}$$

For females, we allow the sexual behaviour of males with females to determine who the females have sex with and how many partners they have. (A more detailed derivation can be found in (Mukandavire, Walker et al. 2018)). The FOI $\Lambda_{i}^{k}$ for the low-risk female population due to their main ($k=m$) and casual ($k=c$) partners is as follows:

$$\Lambda_{1}^{k}=\lambda_{1}\frac{\rho_{1}^{k}}{N_{1}}\sum_{j=2,3,5,6} {[\beta}_{xv}\left( 1-\varepsilon\pi_{1jv}^{k} \right)\Psi_{1jv}^{k}+\beta_{xa}\left( 1-\varepsilon\pi_{1ja}^{k} \right)\Psi_{1ja}^{k}]n_{j}^{k}N_{j}B_{j}$$

With $B_{j}$as the HIV prevalence of the population they are having sex with, which also accounts for the cofactors that increase or decrease HIV transmission risk due to the HIV acute phase $E_{j}$ ($\nu_{E})$ or the pre-AIDS and AIDS stages ($\nu_{A})$, or if on HIV treatment $T_{j}$ ($\alpha)$, such that

$$B_{j}=\frac{\nu_{E}E_{j}+I_{j}+\nu_{A}P_{j}+\left( 1-\alpha\right)(I_{j}^{t}+\nu_{A}P_{j}^{t}+\nu_{A}A_{j}^{t})}{N_{j}}$$

Where $N_{j}$ is the total population for each sub-group $j$.

The FOI for the low-risk male population due to their main $(k=m$) and casual ($k=c$) partners is

$$\Lambda_{2}^{k}=\lambda_{2}{(1-\vartheta\xi)n}_{2}^{k}\sum_{j=1 or 4} {[\beta}_{yv}\left( 1-\varepsilon\pi_{2jv}^{k} \right)\Psi_{2jv}^{k}+\beta_{ya}\left( 1-\varepsilon\pi_{2ja}^{k} \right)\Psi_{2ja}^{k}]\rho_{j}^{k}B_{j}$$

The force of infection for clients due to their main, casual ($k=m,c$) and commercial partners are given as:

$$\Lambda_{3}^{k}=\lambda_{3}(1-\vartheta\xi)n_{3}^{k}\sum_{j=1 or 4} {[\beta}_{yv}\left( 1-\varepsilon\pi_{3jv}^{k} \right)\Psi_{3jv}^{k}+\beta_{ya}\left( 1-\varepsilon\pi_{3ja}^{k} \right)\Psi_{3ja}^{k}]\rho_{j}^{k}B_{j}$$

$$\Lambda_{3}^{co}=\lambda_{3}n_{3}^{co}{[\beta}_{yv}\left( 1-\varepsilon\pi_{34v}^{co} \right)(1-p_{a}^{co})+\beta_{ya}\left( 1-\varepsilon\pi_{34a}^{c} \right)p_{a}^{co}](1-\vartheta\xi)B_{4},$$

Where $p_{a}^{co}$ is the proportion of commercial sex acts that are anal.

The FOI for female sex workers is similarly

$$\Lambda_{4}^{k}=\lambda_{4}\frac{\rho_{4}^{k}}{N_{4}}\sum_{j=2,3,5,6} {{[\beta}_{xv}\left( 1-\varepsilon\pi_{4jv}^{k} \right)\Psi_{4jv}^{k}+\beta_{xa}\left( 1-\varepsilon\pi_{4ja}^{k} \right)\Psi_{4ja}^{k}]n}_{j}^{k}N_{j}B_{j}$$

$$\Lambda_{4}^{co}=\lambda_{4}{n_{4}^{co}[\beta}_{xv}\left( 1-\varepsilon\pi_{43v}^{co} \right)(1-p_{a}^{co})+\beta_{xa}\left( 1-\varepsilon\pi_{43a}^{c} \right)p_{a}^{co}]B_{3}$$

Men who have sex with men are assumed to have main and casual partnerships with women from the low risk female population and FSWs as well as other MSM. The FOI for young MSM due to their main and casual ($k=m,c$) partnerships with females is:

$$\Lambda_{5}^{k}=\lambda_{5}(1-\vartheta\xi)n_{5}^{k}\sum_{j=1 or 4} {[\beta}_{yv}\left( 1-\varepsilon\pi_{5jv}^{k} \right)\Psi_{5jv}^{k}+\beta_{ya}\left( 1-\varepsilon\pi_{5ja}^{k} \right)\Psi_{5ja}^{k}]\rho_{j}^{k}B_{j}$$

For MSM sexual intercourse with their male sexual partners, we denote the receptive AI and insertive AI HIV transmission probability as $\beta_{jrec}$ and $\beta_{jins}$ respectively where $j=5,6$ and subscript *rec* and *ins* are for receptive and insertive anal intercourse between MSM. The asterisk (*) show parameters related to MSM with their male partners. Then, the FOI for young MSM due to their main and casual ($k=m,c$) partnerships with other MSM is:

$$\Lambda_{5*}^{k}=\lambda_{5}\frac{{(\beta}_{5rec}+\beta_{5ins})}{2}(1-\frac{\vartheta\xi}{2})n_{5*}^{k}\sum_{j=5 or 6} \left( 1-\varepsilon\pi_{5j*}^{k} \right){(\Psi}_{5j*}^{k})\rho_{j*}^{k}B_{j}$$

Where $\rho_{5*}^{k}$is the probability of mixing to form MSM male sexual partnerships with young or older MSM, and is given by $\rho_{5*}^{k}=n_{5*}^{k}N_{5}/(n_{5*}^{k}N_{5}+n_{6*}^{k}N_{6})$ and $\rho_{6*}^{k}=n_{6*}^{k}N_{6}/(n_{5*}^{k}N_{5}+n_{6*}^{k}N_{6})$

Similarly, the FOI for older MSM due to their female main and casual ($k=m,c$) partners

$$\Lambda_{6}^{k}=\lambda_{6}(1-\vartheta\xi)n_{6}^{k}\sum_{j=1 or 4} {[\beta}_{yv}\left( 1-\varepsilon\pi_{6jv}^{k} \right)\Psi_{6jv}^{k}+\beta_{ya}\left( 1-\varepsilon\pi_{6ja}^{k} \right)\Psi_{6ja}^{k}]\rho_{j}^{k}B_{j}$$

And due to their main and casual ($k=m,c$) partnerships with other MSM

$$\Lambda_{6*}^{k}=\lambda_{6}\frac{{(\beta}_{6rec}+\beta_{6ins})}{2}(1-\frac{\vartheta\xi}{2})n_{6*}^{k}\sum_{j=5 or 6} {\left( 1-\varepsilon\pi_{6j*}^{k} \right){(\Psi}_{6j*}^{k})\rho}_{j*}^{k}B_{j}$$

# Condom use assumptions

*Low risk male and female – main partners*

Analyses of 3 national studies show that condoms were used by ~6-10% of individuals at sexual debut during the 1980s(Kincaid, Babalola et al. 2014). A study among women attending antenatal clinic in Free state found that condoms were worn with 15.1% of all partners (RHRU 2000a). Another study of women attending a family planning clinic in Gauteng had respondents reporting condom use with 44.8% of all partners in the last 4 weeks (RHRU 2000b). Men attending clinics in Johannesburg reported 17.5% and 14.4% used with main and regular partners in the last 4 weeks (RHRU 2000c). Another study among a rural adult population in South Africa in 2003 showed that 23.1% used condom in their last sex act with a regular partner (Peltzer, Mashego et al. 2003). Condom use in last sex with regular partner was 38.8-43.7% and 33.3-40.5% in the 2005 (Human Sciences Research Council) and 2008 (Human Sciences Research Council) HSRC surveys for males and females, respectively. Quaife et al., 2016 reports condom use of 61.6-74.7% for males and 36.0-50.0% for females with regular partners in the last sex act although the male data seems too high. In the model, we assumed condom use for VI in the low risk population with main partners increased from 0-10% in 1985 to 7.5-23% in 2000, then 16-50% by 2008, and remaining constant thereafter. We assumed half lower bounds in the trend (as given) to account for possible reporting bias and assumed same condom use for males and females.

Condom use for AI among main partners is assumed to be the same as that for VI for these partnership types. A recent systematic review found that in South Africa, condom use during AI was similar to that for VI; among general‐risk populations, the fraction of AI and VI acts that were unprotected was 27.0–53.6% and 26.9–57.0%, respectively (Owen, Elmes et al. 2017).

*Low risk male and female – casual partners*

Early on, men attending STI clinics in Johannesburg reported 8.9% condom use with casual partners in the last 4 weeks (RHRU 2000c), while women attending a family planning clinic in Gauteng reported condom use with 44.8% of all partners in the last 4 weeks (RHRU 2000b). More recently, males and females report condom use in last VI with casual partner of 82.1% (69.4-94.6%) and 75.0% (36.3-100%), respectively (Quaife, Eakle et al. 2016). In 2005, condom use with casual partners for male and female was 60.1-72.2% and 30.7-47.9%, respectively (Human Sciences Research Council), whereas it was 20.2-59.4% and 43.7-78.9%, respectively, during the 2008 survey (Human Sciences Research Council). For casual partners, the model assumes that condom use for VI increases to 4.5-45% in 2000, assuming a similar scale-up as for main partnerships over 1985-2000, and to 10.1-78.9% by 2005, remaining constant thereafter. The Quaife data was assumed to not be representative. We incorporated bias by incorporating half the lower bound in these estimates.

As for main partnerships, condom use for AI among casual partners is assumed to be the same as that for VI for these partnership types based on a recent systematic review (Owen, Elmes et al. 2017).

*FSW and clients – commercial sex*

A cross sectional survey carried out among FSW between 1996 and 1997 in Johannesburg, SA (Dunkle, Beksinska et al. 2005) reported that 34.6-65.6% of the FSW used condom with commercial partners all the time and about 50% in 50-75% of the time. A cross sectional survey of FSW in a semi-urban area of South Africa in Tzaneen and Phalaborwa, Limpopo Province carried out between 2001/02 showed that 41.1-80% of FSW used condoms with paying clients every time (Peltzer, Seoka et al. 2004). A national survey in 2008 found that condom use reported by males was 64.7% (38.3-85.8%) and females was 38.5% ( 20.2-59.4%) in their last sex act with commercial partners (of those who reported commercial partners in the last 12 months) (Human Sciences Research Council). Recent FSW surveys have shown that condom use is now high with commercial partners with 93.0-98.6% of FSW reporting always using condoms with clients (Quaife, Eakle et al. 2016), 84.7-87.7% using condoms at last paid sex act (SAHMS-FSW 2014) and 83.9-98.8% using condoms with new and regular clients in last sex act (PEFSW 2015, Rao, Baral et al. 2016). In a recent (2017-2018) survey in Port Elisabeth, 72.9% (95%CI: 69.0-76.5) and 43.0% (95% CI: 33.2-53.4) of clients reported using a condom at last commercial VI and AI act, respectively. In a recent (2018) survey in Klerksdorp, 91.6% (95%CI: 84.6-96.1) of clients reported using a condom at last commercial sex act. In the model, for VI, we assumed an initial condom use of 0-20% between 1985 and 1990, increasing to 30-90% in 1997 (lower bound from Dunkle data - 34.6%+50%*50% which is 60% and then halved because of likely reporting bias), then to 30-98.8% in 2008 and constant thereafter based on FSW and client surveys and data from general population survey in 2008. We also assumed that condom use did not decrease over this period. For anal sex, we assumed 0.5-1 times the level of condom use as VI over time due to client data and data that 43% (30.2-56.8%) of FSW (n=58) (PEFSW 2015, Rao, Baral et al. 2016) reported condom use at last AI with new clients and 82% (66.5-92.5%, n=39) reported condom use at last AI with regular clients – these are lower than the condom use estimates these FSW report for VI in commercial sex.

*FSW and clients – non-commercial sex*

Data suggests condom use of FSW with main partners was very low in 2000 (5-15%) (RHRU 2000/01), with another survey having 66% of FSW reporting not using a condom with the last non-paying partner in 2001/02 (Peltzer, Seoka et al. 2004). A moderate condom use was reported by FSW in 2013 (SAHMS 2013) of 33.7-41.7% in last vaginal sex act and another FSW survey had condom use of 26.3-44.1% for VI with long term partners (PEFSW 2015, Rao, Baral et al. 2016). In the model, we therefore assumed that condom use during VI with main partners was 2.5-33% in 2000, assuming a similar scale-up to condom use among low-risk females over 1985-2000, increasing to 13-44.1% by 2013 and remaining stable thereafter. We halved the lower bound to account for possible reporting bias. Based on levels of condom use for FSW with their main (26-44%) and casual (37-64%) partners from a recent FSW survey (PEFSW 2015), we assume condom use for casual partners is 1.25-1.75 times that of main partners. For anal sex with main partners, all 14 FSW used condom for last anal sex with main partner in one survey (Quaife, Eakle et al. 2016), whereas out of the 10 FSW who responded in another survey, only 1 used condom during last AI with their long term partner (PEFSW 2015). Data is very limited, so in the model we assumed the same level of condom use for anal sex as for vaginal sex for casual and main partnership sex acts.

In a recent (2017-2018) survey in Port Elisabeth, clients report using condoms in 34.1% (95%CI: 29.5-38.7%) and 73.2% (95%CI: 68.0-78.3) of their vaginal sex acts with main and casual partners in the past 30 days, respectively. In a recent (2018) survey in Klerksdorp, clients report using condoms 37.0% (95%CI: 19.4%-57.6%) and 76.6% (95%CI: 64.3-86.2) at their last sex act with main and casual partners, respectively. We take the upper and lower bounds of these estimates and incorporated bias by incorporating half the lower bound in these estimates. For clients’ condom use with main and casual partners, we assume that condom use is scaled up to 9.7-57.6% and 32.15-86.2, respectively, following the same trend as for low risk males. As was the case for FSW, data is very limited, so in the model we assumed the same level of condom use for anal sex as for vaginal sex for casual and main partnership sex acts; only 17 and 18 participants provide data on condom use for anal sex with main and casual partners, respectively.

*MSM – male main and casual partnerships*

In 2004-5, 58.5% (95% CI: 50.1-66.6) of MSM in Gauteng province reported any unprotected anal sex in the past 6 months(Lane, Shade et al. 2008). In 2008, MSM in Soweto, Johannesburg, reported that amongst male partners in the past 6 months, unprotected anal intercourse typically accounted for 24% of same-sex anal intercourse sex acts with a given partner (Arnold, Struthers et al. 2013). Another 2008 study found similarly low levels of condom use among MSM in Pretoria, where 33% (95% CI: 27.5-38.9) of MSM reported using condoms during the last anal sex act (Knox, Reddy et al. 2013). In 2008, 51.5% (95%CI: 45.6-57.5) of MSM participating in the Johannesburg/eThekwini Men’s Study reported any unprotected anal sex in the past year (Rispel, Metcalf et al. 2011a). In 2009, 6.5% of MSM in Cape Town reported consistent condom use with all sexual partners, 52.4% reported always wearing condoms with male sexual partners and 39.5% reported always wearing condoms with female sexual partners (Baral, Burrell et al. 2011). In 2010, 37.8% (95%CI: 33.6-42.1%) of internet using MSM (recruited on Facebook) reported using a condom at last sex act (Stephenson, de Voux et al. 2011). In another 2010 study, 55.2% of MSM in Cape Town reported any unprotected anal sex in the last 6 months (Tucker, Liht et al. 2013, Tucker, Liht et al. 2014). In 2012, 62% of a small sample of MSM in Cape Town and Port Elisabeth (n=34) reported always using condoms with their most recent anal sex partner (Siegler, Voux et al. 2014). In 2011-13, 47.1% (95% CI: 42.5-51.7) of MSM reported unprotected anal sex in the past 3 months (Knox, Reddy et al. 2017).

Studies conducted from 2012/13 onwards consistently find high levels of condom use at last sex act. In the Mpumalanga Men’s study (2012-2013), 85.1% (95% CI: 78.2-90.7) and 63.7% (95%CI 55.9-70.1) of MSM in Gert Sibande and Ehlanzeni, respectively, reported consistent condom use with their last 5 male partners (Lane, Osmand et al. 2014). In the South Africa Marang Men’s project (2012-13), 80.7% (95%CI:74.0-88.1), 84.6% (95%CI: 78.8-89.2), and 80.5% (95%CI: 74.2-86.7) of MSM in Durban, Cape Town and Johannesburg, respectively, reported condom use in the last sex act (Cloete, Jooste et al. 2014). In 2014-2015, an internet survey of MSM found that 60.0% (95%CI: 55.1-64.8) of MSM reported unprotected anal sex in the past 6 months (Hugo, Stall et al. 2016). In the Sibanye Health Project (2015), 80.2% (95%CI: 71.8-88.7%) and 69.5% (95%CI: 61.1-77.9%) of MSM in Cape Town and Port Elisabeth, respectively, reported using condoms in the last sex act with a main male partner. In the same survey, similar rates of condom use were reported for casual partners; 76.9% (95%CI: 67.5-86.2) and 80.3% (95%CI: 73.5-87.3) of MSM in Cape Town and Port Elisabeth, respectively, reported using condoms) in the last sex act with a casual male partner. Hence, we assume that condom use for homosexual sex is the same for sex with main and casual partners. We incorporated bias by halving the lower bound in all these estimates. In the model we assume that condom use between MSM is scaled up to 12-54.4% in 2008, following the same trend as low-risk men over 1985-2008, and increases to 28-90.7% in 2014. Condom use is then assumed to remain stable.

*MSM and female partners – main and casual partnerships*

Little data is available on condom use between MSM and their female partners. Estimates from the Sibanye Health Project (2015) suggest that MSM in Port Elisabeth and Cape Town have high condom use with their female partners: 71.4% (n=7; 95%CI: 21.5-95.8) and 59.3% (n=27; 95%CI: 39.0-76.8) of MSM report condom use at their last anal and vaginal sex with a main female partner and 76.2% (n=9; 95%CI: 51.7-90.5) and 77.8% (n=21; 95%CI: 33.0-96.1%) of MSM report condom use at their last anal and vaginal sex with a casual female partner. We therefore assume that condom use for MSM with their female partners is the same as with their male partners.

## Male circumcision coverage assumptions

The model assumes 38.0% (32.4-42.0%) of men are circumcised between 1985-2002 because of tribal groups like the Xhosa who carry out traditional circumcision (Human Sciences Research Council , Mayatula and Mavundla 1997, Meissner and Buso 2007). It was assumed to increase from 2002 to 41% (36.8-43.3%) by 2008 (Human Sciences Research Council), to 46.4% (44.1-48.8%) by 2012 (Human Sciences Research Council) and to 61.6% (59.3-63.9) by 2017 (Human Sciences Research Council). We assume stable thereafter. We assume that all male risk groups (i.e. low risk males, MSM and clients) have the same levels of circumcision.

# Model Calibration

The model was calibrated using an approximate Bayesian computation Sequential Monte Carlo (ABC SMC) method,(Toni, Welch et al. 2009) which accounts for uncertainty in the calibration data and parameters and ranks different model runs by their goodness of fit. The ABC method was used to calibrate the model to total population and KP size estimates, KP size estimates (FSW in 2013, MSM in 2005), ART coverage levels among all adults in 2010 and in different sub-groups in 2018, HIV incidence estimates from the HSRC surveys for the adult population, different groups: adult males and females (15-49 years) from the HSRC surveys (2005, 2012 and 2017); FSWs in 2000 (38.6-58.8%) and 2016 (45.5-68.0%); young MSM in 2009 (13.2-56.5%) and all MSM in 2016 (20.0-58.2%). Calibration ranges were defined for each estimate to be the 95% confidence interval (CI) of an estimate. For HIV prevalence among FSW and MSM, where there were multiple estimates at similar timepoints, the calibration range was defined to be the minimum lower 95%CI to the maximum upper 95%CI across these estimates. Goodness of fit for each parameter set was then defined as the sum of the absolute differences (defined as 0 if model projections lie within the calibration range) on the log scale between the lower or upper (whichever is closer to the model projections) value of the calibration ranges and the corresponding model projections.

The ABC SMC begins with 10,000 parameter sets sampled from prior distributions using Latin Hypercube sampling, which are then successively perturbed to improve their goodness of fit. At each subsequent iteration, parameter sets are resampled from the previous iteration and perturbed, until 10,000 parameter sets are obtained iteratively so that which fit the data at least as well as the best 75% parameter sets from the previous iteration. The ABC continues each time the parameter sets better fit the data, until successive iterations no longer improve the goodness of the fits. The ABC routine produced a set of 10,000 baseline model fits which were used to give the median and 95% credibility intervals (95%CrI; 2.5^th^ to 97.5^th^ percentile range) for all model projections.

# Model Cross-validation

In all model runs, HIV prevalence among clients falls within the range of prevalences observed in Port Elizabeth and Klerksdorp, whilst 78.5% of runs fall within the range of the previous HIV incidence estimate among FSW and 46.1% of runs fall within the range of all 3 HIV incidence estimates among MSM.

# References

Abuelezam, N. N., A. W. McCormick, T. Fussell, A. N. Afriyie, R. Wood, V. DeGruttola, K. A. Freedberg, M. Lipsitch and G. R. Seage, 3rd (2016). "Can the Heterosexual HIV Epidemic be Eliminated in South Africa Using Combination Prevention? A Modeling Analysis." Am J Epidemiol **184**(3): 239-248.

Andersson, K., R. Van Niekerk, L. Niccolai, O. Mlungwana, I. Holdsworth, M. Bogoshi, J. McIntyre, G. Gray and E. Vardas (2009). "Sexual risk behaviour of the first cohort undergoing screening for enrolment into Phase I/II HIV vaccine trials in South Africa." International journal of STD & AIDS **20**(2): 95-101.

Arnold, M. P., H. Struthers, J. McIntyre and T. Lane (2013). "Contextual correlates of per partner unprotected anal intercourse rates among MSM in Soweto, South Africa." AIDS & Behavior **17 Suppl 1**: S4-11.

Baggaley, R. F., R. G. White and M. C. Boily (2010). "HIV transmission risk through anal intercourse: systematic review, meta-analysis and implications for HIV prevention." Int J Epidemiol **39**(4): 1048-1063.

Baral, S., E. Burrell, A. Scheibe, B. Brown, C. Beyrer and L. G. Bekker (2011). "HIV risk and associations of HIV infection among men who have sex with men in peri-urban Cape Town, South Africa." BMC Public Health **11**: 766.

Barnabas, R. V., H. van Rooyen, E. Tumwesigye, P. M. Murnane, J. M. Baeten, H. Humphries, B. Turyamureeba, P. Joseph, M. Krows, J. P. Hughes and C. Celum (2014). "Initiation of antiretroviral therapy and viral suppression after home HIV testing and counselling in KwaZulu-Natal, South Africa, and Mbarara district, Uganda: a prospective, observational intervention study." Lancet HIV **1**(2): e68-e76.

Barth, R. E., H. A. Tempelman, R. Moraba and A. I. Hoepelman (2011). "Long-Term Outcome of an HIV-Treatment Programme in Rural Africa: Viral Suppression despite Early Mortality." AIDS Res Treat **2011**: 434375.

Behanzin, L., S. Diabate, I. Minani, C. M. Lowndes, M. C. Boily, A. C. Labbe, S. Anagonou, D. M. Zannou, A. Buve and M. Alary (2013). "Assessment of HIV-related risky behaviour: a comparative study of face-to-face interviews and polling booth surveys in the general population of Cotonou, Benin." Sex Transm Infect **89**(7): 595-601.

Bekker, L. G., L. Johnson, F. Cowan, C. Overs, D. Besada, S. Hillier and W. Cates, Jr. (2015). "Combination HIV prevention for female sex workers: what is the evidence?" Lancet **385**(9962): 72-87.

Boily, M. C., R. F. Baggaley, L. Wang, B. Masse, R. G. White, R. J. Hayes and M. Alary (2009). "Heterosexual risk of HIV-1 infection per sexual act: systematic review and meta-analysis of observational studies." Lancet Infect Dis **9**(2): 118-129.

Carael, M., E. Slaymaker, R. Lyerla and S. Sarkar (2006). "Clients of sex workers in different regions of the world: hard to count." Sex Transm Infect **82 Suppl 3**: iii26-33.

Cloete, A., S. Jooste, M. Mabaso, L. Simbayi, T. Rehle and P. Naidoo (2014). "The South African Marang men's project: HIV bio-behavioural surveys conducted among men who have sex with men in Cape Town, Durban and Johannesburg using respondent driven sampling."

Coetzee, J., G. Hunt, M. Jaffer, K. Otwombe, L. Scott, A. Bongwe, J. Ledwaba, S. Molema, R. Jewkes and G. E. Gray (2017). "HIV-1 viraemia and drug resistance amongst female sex workers in Soweto, South Africa: A cross sectional study." PloS one **12**(12): e0188606.

Coetzee, J., R. Jewkes and G. E. Gray (2017). "Cross-sectional study of female sex workers in Soweto, South Africa: factors associated with HIV infection." PloS one **12**(10): e0184775.

Dunkle, K. L., M. E. Beksinska, V. H. Rees, R. C. Ballard, Y. Htun and M. L. Wilson (2005). "Risk factors for HIV infection among sex workers in Johannesburg, South Africa." Int J STD AIDS **16**(3): 256-261.

Dunkle, K. L., R. K. Jewkes, D. W. Murdock, Y. Sikweyiya and R. Morrell (2013). "Prevalence of consensual male-male sex and sexual violence, and associations with HIV in South Africa: a population-based cross-sectional study." PLoS Medicine / Public Library of Science **10**(6): e1001472.

Eakle, R., G. B. Gomez, N. Naicker, R. Bothma, J. Mbogua, M. A. Cabrera Escobar, E. Saayman, M. Moorhouse, W. D. F. Venter, H. Rees and T. D. P. Team (2017). "HIV pre-exposure prophylaxis and early antiretroviral treatment among female sex workers in South Africa: Results from a prospective observational demonstration project." PLoS Med **14**(11): e1002444.

Fatti, G., E. Mothibi, G. Meintjes and A. Grimwood (2014). "Antiretroviral treatment outcomes amongst older adults in a large multicentre cohort in South Africa." PloS one **9**(6): e100273.

Gomez, G. B., R. Eakle, J. Mbogua, G. Akpomiemie, W. D. Venter and H. Rees (2016). "Treatment And Prevention for female Sex workers in South Africa: protocol for the TAPS Demonstration Project." BMJ Open **6**(9): e011595.

Hollingsworth, T. D., R. M. Anderson and C. Fraser (2008). "HIV-1 transmission, by stage of infection." J Infect Dis **198**(5): 687-693.

Huerga, H., F. Shiferie, E. Grebe, R. Giuliani, J. B. Farhat, G. Van-Cutsem and K. Cohen (2017). "A comparison of self-report and antiretroviral detection to inform estimates of antiretroviral therapy coverage, viral load suppression and HIV incidence in Kwazulu-Natal, South Africa." BMC infectious diseases **17**(1): 653.

Hughes, J. P., J. M. Baeten, J. R. Lingappa, A. S. Magaret, A. Wald, G. de Bruyn, J. Kiarie, M. Inambao, W. Kilembe, C. Farquhar, C. Celum and H. S. V. H. I. V. T. S. T. Partners in Prevention (2012). "Determinants of per-coital-act HIV-1 infectivity among African HIV-1-serodiscordant couples." J Infect Dis **205**(3): 358-365.

Hugo, J. M., R. D. Stall, K. Rebe, J. E. Egan, G. Jobson, G. De Swardt, H. Struthers and J. A. McIntyre (2016). "Knowledge, Attitudes and Beliefs regarding Post Exposure Prophylaxis among South African Men who have Sex with Men." AIDS & Behavior **20**(Suppl 3): 350-356.

Human Sciences Research Council "The Fifth South African National HIV Prevalence, Incidence, Behaviour and Communication Survey, 2017."

Human Sciences Research Council South African HIV/AIDS, Behavioural Risks, Sero-status, and Mass Media Impact Survey (SABSSM) 2002: Adult and youth data - All provinces. [Data set]. SABSSM 2002 Adult-youth. Version 1.0. Pretoria South Africa: Human Sciences Research Council [producer] 2002, Human Sciences Research Council [distributor] 2011. <http://dx.doi.org/doi:10.14749/1400830395>.

Human Sciences Research Council South African National HIV Prevalence, HIV Incidence, Behaviour and Communication Survey (SABSSM) 2005: Adult and youth data - All provinces. [Data set]. SABSSM 2005 Adult-youth. Version 1.0. Pretoria South Africa: Human Sciences Research Council [producer] 2005, Human Sciences Research Council [distributor] 2011. <http://dx.doi.org/doi:10.14749/1400830455>.

Human Sciences Research Council South African National HIV Prevalence, HIV Incidence, Behaviour and Communication Survey (SABSSM) 2008: Adult - All provinces. [Data set]. SABSSM 2008 Adult. Version 1.0. Pretoria South Africa: Human Sciences Research Council [producer] 2009, Human Sciences Research Council [distributor] 2014. <http://dx.doi.org/doi:10.14749/1434098373>.

Human Sciences Research Council South African National HIV Prevalence, HIV Incidence, Behaviour and Communication Survey (SABSSM) 2012: Adult - All provinces. [Data set]. SABSSM 2012 Adult. Version 1.0. Pretoria South Africa: Human Sciences Research Council [producer] 2012, Human Sciences Research Council [distributor] 2016. <http://dx.doi.org/doi:10.14749/1500530684>.

Jean, K., A. Puren, E. Cutler, B. Singh, J. Bouscaillou, R. Rain-Taljaard, D. Taljaard, E. Gouws, P. Lissouba and D. A. Lewis (2016). "Level of viral suppression and the cascade of HIV care in a South African semi-urban setting in 2012." Aids **30**(13): 2107-2116.

Jewkes, R., K. Dunkle, M. Nduna, J. Levin, N. Jama, N. Khuzwayo, M. Koss, A. Puren and N. Duvvury (2006). "Factors associated with HIV sero-positivity in young, rural South African men." International Journal of Epidemiology **35**(6): 1455-1460.

Johansson, K. A., B. Robberstad and O. F. Norheim (2010). "Further benefits by early start of HIV treatment in low income countries: survival estimates of early versus deferred antiretroviral therapy." AIDS Res Ther **7**(1): 3.

Johnson, L. F., C. Chiu, L. Myer, M.-A. Davies, R. E. Dorrington, L.-G. Bekker, A. Boulle and G. Meyer-Rath (2016). "Prospects for HIV control in South Africa: a model-based analysis." Global health action **9**(1): 30314.

Johnson, L. F., P. Mulongeni, A. Marr and T. Lane (2018). "Age bias in survey sampling and implications for estimating HIV prevalence in men who have sex with men: insights from mathematical modelling." Epidemiol Infect **146**(8): 1036-1042.

Kalichman, S. C., L. Simbayi, D. Cain and S. Jooste (2009). "Heterosexual anal intercourse among community and clinical settings in Cape Town, South Africa." Sexually transmitted infections **85**(6): 411-415.

Kapiamba, G., T. Masango and D. Mphuthi (2016). "Antiretroviral adherence and virological outcomes in HIV-positive patients in Ugu district, KwaZulu-Natal province." African Journal of AIDS Research **15**(3): 195-201.

Kincaid, D. L., S. Babalola and M. E. Figueroa (2014). "HIV communication programs, condom use at sexual debut, and HIV infections averted in South Africa, 2005." JAIDS Journal of Acquired Immune Deficiency Syndromes **66**: S278-S284.

Knox, J., V. Reddy, F. Kaighobadi, D. Nel and T. Sandfort (2013). "Communicating HIV status in sexual interactions: assessing social cognitive constructs, situational factors, and individual characteristics among South African MSM." AIDS & Behavior **17**(1): 350-359.

Knox, J., V. Reddy, T. Lane, D. Hasin and T. Sandfort (2017). "Substance Use and Sexual Risk Behavior Among Black South African Men Who Have Sex With Men: The Moderating Effects of Reasons for Drinking and Safer Sex Intentions." AIDS & Behavior **21**(7): 2023-2032.

Knox, J., H. Yi, V. Reddy, S. Maimane and T. Sandfort (2010). "The fallacy of intimacy: sexual risk behaviour and beliefs about trust and condom use among men who have sex with men in South Africa." Psychology Health & Medicine **15**(6): 660-671.

Kufa, T., T. Lane, A. Manyuchi, B. Singh, Z. Isdahl, T. Osmand, M. Grasso, H. Struthers, J. McIntyre and Z. Chipeta (2017). "The accuracy of HIV rapid testing in integrated bio-behavioral surveys of men who have sex with men across 5 Provinces in South Africa." Medicine **96**(28).

Lane, T., T. Osmand, A. Marr, S. B. Shade, K. Dunkle, T. Sandfort, H. Struthers, S. Kegeles and J. A. McIntyre (2014). "The Mpumalanga Men's Study (MPMS): results of a baseline biological and behavioral HIV surveillance survey in two MSM communities in South Africa." PLoS ONE [Electronic Resource] **9**(11): e111063.

Lane, T., H. F. Raymond, S. Dladla, J. Rasethe, H. Struthers, W. McFarland and J. McIntyre (2011). "High HIV prevalence among men who have sex with men in Soweto, South Africa: results from the Soweto Men's Study." AIDS & Behavior **15**(3): 626-634.

Lane, T., S. B. Shade, J. McIntyre and S. F. Morin (2008). "Alcohol and sexual risk behavior among men who have sex with men in South african township communities." AIDS & Behavior **12**(4 Suppl): S78-85.

Lecher, S. (2016). "Progress with scale-up of HIV viral load monitoring—seven sub-Saharan African countries, January 2015–June 2016." MMWR. Morbidity and mortality weekly report **65**.

Lippman, S. A., T. Lane, O. Rabede, H. Gilmore, Y. H. Chen, N. Mlotshwa, K. Maleke, A. Marr and J. A. McIntyre (2018). "High Acceptability and Increased HIV-Testing Frequency After Introduction of HIV Self-Testing and Network Distribution Among South African MSM." Journal of Acquired Immune Deficiency Syndromes: JAIDS **77**(3): 279-287.

Lippman, S. A., S. B. Shade, A. M. El Ayadi, J. M. Gilvydis, J. S. Grignon, T. Liegler, J. Morris, E. Naidoo, L. M. Prach and A. Puren (2016). "Attrition and opportunities along the HIV care continuum: findings from a population-based sample, North West Province, South Africa." Journal of acquired immune deficiency syndromes (1999) **73**(1): 91.

Mayatula, V. and T. R. Mavundla (1997). "A review on male circumcision procedures among South African blacks." Curationis **20**(3): 16-20.

Mberi, M. N., L. R. Kuonza, N. M. Dube, C. Nattey, S. Manda and R. Summers (2015). "Determinants of loss to follow-up in patients on antiretroviral treatment, South Africa, 2004-2012: a cohort study." BMC Health Serv Res **15**: 259.

McNaghten, A., R. Kearns, A. J. Siegler, N. Phaswana-Mafuya, L.-G. Bekker, R. Stephenson, S. D. Baral, R. Brookmeyer, C. S. Yah and A. J. Lambert (2014). "Sibanye methods for prevention packages program project protocol: pilot study of HIV prevention interventions for men who have sex with men in South Africa." JMIR research protocols **3**(4).

Meissner, O. and D. L. Buso (2007). "Traditional male circumcision in the Eastern Cape--scourge or blessing?" S Afr Med J **97**(5): 371-373.

Mills, E. J., C. Bakanda, J. Birungi, K. Chan, N. Ford, C. L. Cooper, J. B. Nachega, M. Dybul and R. S. Hogg (2011). "Life expectancy of persons receiving combination antiretroviral therapy in low-income countries: a cohort analysis from Uganda." Ann Intern Med **155**(4): 209-216.

Milovanovic, M., M. Jaffer, V. Mbowane, K. Hlongwane, K. Otwombe, J. Buckley, R. Jewkes, G. Gray, H. Hausler, N. Martinson, S. Strathdee and C. J. Male clients of female sex workers in South Africa: Sexual risk behaviour and violence. The 9th SA AIDS Conference 2019, Durban.

Moyo, F., C. Chasela, A. T. Brennan, O. Ebrahim, I. M. Sanne, L. Long and D. Evans (2016). "Treatment outcomes of HIV-positive patients on first-line antiretroviral therapy in private versus public HIV clinics in Johannesburg, South Africa." Clinical epidemiology **8**: 37.

Mukandavire, C., J. Walker, S. Schwartz, M. C. Boily, L. Danon, C. Lyons, D. Diouf, B. Liestman, N. L. Diouf and F. Drame (2018). "Estimating the contribution of key populations towards the spread of HIV in Dakar, Senegal." Journal of the International AIDS Society **21**: e25126.

Muller, E. E., K. Rebe, T. F. Chirwa, H. Struthers, J. McIntyre and D. A. Lewis (2016). "The prevalence of human papillomavirus infections and associated risk factors in men-who-have-sex-with-men in Cape Town, South Africa." BMC Infect Dis **16**(1): 440.

Owen, B. N., J. Elmes, R. Silhol, Q. Dang, I. McGowan, B. Shacklett, E. M. Swann, A. Van der Straten, R. F. Baggaley and M. C. Boily (2017). "How common and frequent is heterosexual anal intercourse among South Africans? A systematic review and meta‐analysis." Journal of the International AIDS Society **20**(1): 21162.

Parry, C., P. Petersen, S. Dewing, T. Carney, R. Needle, K. Kroeger and L. Treger (2008). "Rapid assessment of drug-related HIV risk among men who have sex with men in three South African cities." Drug Alcohol Depend **95**(1-2): 45-53.

PEFSW (2015). Female Sex Workers in Port Elizabeth.

Peltzer, K., T. A. Mashego and M. Mabeba (2003). "Attitudes and practices of doctors toward domestic violence victims in South Africa." Health Care Women Int **24**(2): 149-157.

Peltzer, K., P. Seoka and S. Raphala (2004). "Characteristics of female sex workers and their HIV/AIDS/STI knowledge, attitudes and behaviour in semi-urban areas in South Africa." Curationis **27**(1): 4-11.

Quaife, M., R. Eakle, M. Cabrera, P. Vickerman, M. Tsepe, F. Cianci, S. Delany-Moretlwe and F. Terris-Prestholt (2016). "Preferences for ARV-based HIV prevention methods among men and women, adolescent girls and female sex workers in Gauteng Province, South Africa: a protocol for a discrete choice experiment." BMJ Open **6**(6): e010682.

Ramjee, G., S. S. Karim and A. W. Sturm (1998). "Sexually transmitted infections among sex workers in KwaZulu-Natal, South Africa." Sex Transm Dis **25**(7): 346-349.

Rao, A., S. Baral, N. Phaswana-Mafuya, A. Lambert, Z. Kose, M. McIngana, C. Holland, S. Ketende and S. Schwartz (2016). "Pregnancy Intentions and Safer Pregnancy Knowledge Among Female Sex Workers in Port Elizabeth, South Africa." Obstet Gynecol **128**(1): 15-21.

Rebe, K., D. Lewis, L. Myer, G. de Swardt, H. Struthers, M. Kamkuemah and J. McIntyre (2015). "A Cross Sectional Analysis of Gonococcal and Chlamydial Infections among Men-Who-Have-Sex-with-Men in Cape Town, South Africa." PLoS ONE [Electronic Resource] **10**(9): e0138315.

Rees, H., M. E. Beksinska, K. Dickson-Tetteh, R. Ballard and Y. Htun (2000). "Commercial sex workers in Johannesburg: risk behaviour and HIV status." South African Journal of Science **96**(6).

Rees, K., O. Radebe, C. Arendse, C. Modibedi, H. E. Struthers, J. A. McIntyre and R. P. H. Peters (2017). "Utilization of Sexually Transmitted Infection Services at 2 Health Facilities Targeting Men Who Have Sex With Men in South Africa: A Retrospective Analysis of Operational Data." Sexually Transmitted Diseases **44**(12): 768-773.

RHRU (2000a). "Reproductive Health Research Unit Behavioural Sentinel Survey data report - Antenatal Clinic - Free State; 2000."

RHRU (2000b). "Reproductive Health Research Unit Behavioural Sentinel Survey data report - Clients of family planning - Gauteng.".

RHRU (2000c). "Reproductive Health Research Unit Behavioural Sentinel Survey data report - Male STD clinic attenders - Gauteng.".

Rispel, L. C., C. A. Metcalf, A. Cloete, V. Reddy and C. Lombard (2011a). "HIV prevalence and risk practices among men who have sex with men in two South African cities." Journal of Acquired Immune Deficiency Syndromes: JAIDS **57**(1): 69-76.

Rispel, L. C., C. A. Metcalf, A. Cloete, V. Reddy and C. Lombard (2011b). "HIV prevalence and risk practices among men who have sex with men in two South African cities." J Acquir Immune Defic Syndr **57**(1): 69-76.

Rosen, S., M. Maskew, M. P. Fox, C. Nyoni, C. Mongwenyana, G. Malete, I. Sanne, D. Bokaba, C. Sauls, J. Rohr and L. Long (2016). "Initiating Antiretroviral Therapy for HIV at a Patient's First Clinic Visit: The RapIT Randomized Controlled Trial." PLoS Med **13**(5): e1002015.

SAHMS-FSW (2014). South African Health Monitoring Survey (SAHMS): An Integrated Biological and Behavioural Survey among Female Sex Workers, South Africa 2013-2014.

Sánchez, J., V. G. S. y Rosas, J. P. Hughes, J. M. Baeten, J. Fuchs, S. P. Buchbinder, B. A. Koblin, M. Casapia, A. Ortiz and C. Celum (2011). "Male circumcision and risk of HIV acquisition among men who have sex with men from the United States and Peru." AIDS (London, England) **25**(4): 519.

Sandfort, T. G., T. Lane, C. Dolezal and V. Reddy (2015). "Gender Expression and Risk of HIV Infection Among Black South African Men Who Have Sex with Men." AIDS Behav **19**(12): 2270-2279.

Sandfort, T. G., J. Nel, E. Rich, V. Reddy and H. Yi (2008). "HIV testing and self-reported HIV status in South African men who have sex with men: results from a community-based survey." Sex Transm Infect **84**(6): 425-429.

Schoub, B. D., A. N. Smith, S. Johnson, D. J. Martin, S. F. Lyons, G. N. Padayachee and H. S. Hurwitz (1990). "Considerations on the further expansion of the AIDS epidemic in South Africa--1990." S Afr Med J **77**(12): 613-618.

Shearer, K., A. T. Brennan, M. Maskew, L. Long, R. Berhanu, I. Sanne and M. P. Fox (2014). "The relation between efavirenz versus nevirapine and virologic failure in Johannesburg, South Africa." Journal of the International AIDS Society **17**(1): 19065.

Siegfried, N., M. Muller, J. J. Deeks and J. Volmink (2009). "Male circumcision for prevention of heterosexual acquisition of HIV in men." Cochrane Database Syst Rev(2): CD003362.

Siegler, A. J., A. Voux, N. Phaswana-Mafuya, L. G. Bekker, P. S. Sullivan, S. D. Baral, K. Winskell, Z. Kose, A. L. Wirtz and R. Stephenson (2014). "Elements of Condom-Use Decision Making among South African Men Who Have Sex with Men." Journal of the International Association of Providers of AIDS Care **13**(5): 414-423.

Simbayi, L. C., S. C. Kalichman, S. Jooste, C. Cherry, S. Mfecane and D. Cain (2005). "Risk factors for HIV-AIDS among youth in Cape Town, South Africa." AIDS and Behavior **9**(1): 53-61.

Slabbert, M., F. Venter, C. Gay, C. Roelofsen, S. Lalla-Edward and H. Rees (2017). "Sexual and reproductive health outcomes among female sex workers in Johannesburg and Pretoria, South Africa: Recommendations for public health programmes." BMC Public Health **17**(Suppl 3): 442.

Stephenson, R., A. de Voux and P. S. Sullivan (2011). "Intimate Partner Violence and Sexual Risk-taking among Men Who Have Sex with Men in South Africa." The Western Journal of Emergency Medicine **12**(3): 343-347.

SWPSSES (2013). Estimating the size of the sex worker population in South Africa, 2013. Sex worker population size estimate study (SWPSES).

TB HIV Care (2018). "Viral hepatitis C initiative for key populations in South Africa. Findings summary brief. Vol. 79. Cape Town: TB HIV Care.".

Templeton, D. J., F. Jin, L. Mao, G. P. Prestage, B. Donovan, J. Imrie, S. Kippax, J. M. Kaldor and A. E. Grulich (2009). "Circumcision and risk of HIV infection in Australian homosexual men." Aids **23**(17): 2347-2351.

Todd, J., J. R. Glynn, M. Marston, T. Lutalo, S. Biraro, W. Mwita, V. Suriyanon, R. Rangsin, K. E. Nelson, P. Sonnenberg, D. Fitzgerald, E. Karita and B. Zaba (2007). "Time from HIV seroconversion to death: a collaborative analysis of eight studies in six low and middle-income countries before highly active antiretroviral therapy." AIDS **21 Suppl 6**: S55-63.

Toni, T., D. Welch, N. Strelkowa, A. Ipsen and M. P. Stumpf (2009). "Approximate Bayesian computation scheme for parameter inference and model selection in dynamical systems." J R Soc Interface **6**(31): 187-202.

Tsondai, P. R., L. S. Wilkinson, A. Grimsrud, P. T. Mdlalo, A. Ullauri and A. Boulle (2017). "High rates of retention and viral suppression in the scale‐up of antiretroviral therapy adherence clubs in Cape Town, South Africa." Journal of the International AIDS Society **20**: 21649.

Tucker, A., J. Liht, G. de Swardt, G. Jobson, K. Rebe, J. McIntyre and H. Struthers (2013). "An exploration into the role of depression and self-efficacy on township men who have sex with men's ability to engage in safer sexual practices." AIDS Care **25**(10): 1227-1235.

Tucker, A., J. Liht, G. de Swardt, G. Jobson, K. Rebe, J. McIntyre and H. Struthers (2014). "Homophobic stigma, depression, self-efficacy and unprotected anal intercourse for peri-urban township men who have sex with men in Cape Town, South Africa: a cross-sectional association model." AIDS Care **26**(7): 882-889.

UNAIDS-AIDSinfo UNAIDS-AIDSinfo. Available at <http://aidsinfo.unaids.org/>.

United Nation, Department of Economic and Social Affairs and P. Division (2015). UN World Population Prospects: The 2015 Revision.

Weller, S. and K. Davis (2001). "Condom effectiveness in reducing heterosexual HIV transmission." Cochrane Database Syst Rev(3): CD003255.

Zanoni, B. C., T. Sibaya, C. Cairns, S. Lammert and J. E. Haberer (2017). "Higher retention and viral suppression with adolescent-focused HIV clinic in South Africa." PloS one **12**(12): e0190260.

Zhang, Y., J. M. Fogel, X. Guo, W. Clarke, A. Breaud, V. Cummings, E. L. Hamilton, A. Ogendo, N. Kayange and R. Panchia (2018). "Antiretroviral drug use and HIV drug resistance among MSM and transgender women in sub-Saharan Africa." Aids **32**(10): 1301-1306.

## Supplementary Table 1. Details of surveys used to parameterise and calibrate the model . FSW denotes female sex workers; MSM denotes men who have sex with men.

| Year | How sampled/recruited | Sample size | Setting | HIV prevalence % (95%CI) | Reference |
| --- | --- | --- | --- | --- | --- |
| FSW | | | | | |
| 1997/98 | Venue/street based | 295 | Hillbrow | 46.4% (40.6-52.3) | (Dunkle, Beksinska et al. 2005) |
| 1996/97 | Recruited from five truck stops | 145 | KwaZulu-Natal | 50.3% (41.2-58.7) | (Ramjee, Karim et al. 1998) |
| 2000 | Outreach workers | 247 | Hillbrow, Johannesburg | 44.9% (38.6-51.4) | (Rees, Beksinska et al. 2000) |
| 2013/14 | Respondent driven sampling | 764  650  766 | Johannesburg  Cape Town  Durban  Overall | 71.8% (56.5-81.2)  39.5% (30.1-49.8)  53.5% (37.5-65.6)  58.5% (56.4-60.6) | (SAHMS-FSW 2014) |
| 2014/15 | Peer educators recruited FSW who can then access health HIV and sexual reproductive health services. Retrospective analysis | 1422  408 | Johannesburg (1364 tested)  Pretoria (399 tested) | 42.2% (39.5-44.8)  52.9% (47.9-57.9) | (Slabbert, Venter et al. 2017) |
| 2014/15 | Respondent driven sampling | 410 | Port Elizabeth and surrounding areas | 61.5% (54.1-68.0) | (PEFSW 2015, Rao, Baral et al. 2016) |
| 2015/16 | Venue/street based | 692 | Pretoria and Inner Johannesburg | 49.2% (45.5-53.1) | (Gomez, Eakle et al. 2016, Eakle, Gomez et al. 2017) |
| 2016 | Respondent driven sampling | 508 | Soweto | 53.6% (47.5-59.9) | (Coetzee, Jewkes et al. 2017) |
| 2016/17 | Opportunity based sampling whilst accessing community-based HIV services for Key populations | 1531 | Cape Town, Durban, Mthatha, Pietermaritzburg, Port Elizabeth | 46.6% (44.0-49.1) | (TB HIV Care) |
| Clients | | | | | |
| 2017/18 | Venue/street based (time location sampling) | 600 | Port Elizabeth | 14.0% (11.4-17.0) | Unpublish-ed data |
| 2018 | Recruited by FSW | 115 | Klerksdorp | 29.8% (21.6-39.1) | (Milovanovic, Jaffer et al.) |
| MSM | | | | | |
| Unknown | Internet-based | 824 | National | Not available | (Stephenson, de Voux et al. 2011) |
| 1988-1990 | Attendees of STD and family planning clinics | 86 | Johannesburg | 18.6% (11.0-28.4) | (Schoub, Smith et al. 1990) |
| 2003/2005 | Event/network based and internet-based | 1,045 | Gauteng, KwaZulu-Natal and Western Cape | Not available | (Sandfort, Nel et al. 2008) |
| 2016/17 | Opportunity based sampling whilst accessing community-based HIV services for Key populations | 747 | Cape Town, Johannesburg, Pretoria | 42.8% (39.3-46.5) | (TB HIV Care) |
| 2015/16 | Respondent driven sampling | 545  525  476  359 | Johannesburg  Bloemfontein  Mafikeng  Polokwane | 43.4%  17.3%  14.6%  22.4% | (Kufa, Lane et al. 2017, Johnson, Mulongeni et al. 2018) |
| 2014/15 | Internet-based survey | 408 | National | Not available | (Hugo, Stall et al. 2016) |
| 2012/13 | Respondent driven sampling | 307  298 | Gert Sibande  Ehlanzeni | 28.3% (21.1–35.3)  13.7% (9.1–19.6) | (Lane, Osmand et al. 2014) |
| 2004/5 | Chain-referral and venue-based recruitment | 199 | Gauteng province | Not available | (Lane, Shade et al. 2008) |
| 2011-13 | Respondent driven sampling of black MSM | 480 | Pretoria | 30.1% | (Sandfort, Lane et al. 2015, Knox, Reddy et al. 2017) |
| 2014-16 | Clients of primary health care facilities | 5,796; 1,107 MSM | Johannesburg | 58.4% (55.4-61.3) | (Rees, Radebe et al. 2017) |
| 2005 | Street intercepts and snowball sampling | 37 | Cape Town, Durban, Pretoria | 35.1% (20.2-52.5) | (Parry, Petersen et al. 2008) |
| 2008 | Respondent driven sampling | 378 | Soweto | 13.2% (12.4-13.9) | (Lane, Raymond et al. 2011, Arnold, Struthers et al. 2013) |
| 2008 | Event/venue based. | 300 | Pretoria | Not available | (Knox, Yi et al. 2010, Knox, Reddy et al. 2013) |
| 2008 | Household survey | 73 MSM | Eastern Cape and KwaZulu-Natal provinces | 27.4% (17.6-40.0) | (Dunkle, Jewkes et al. 2013) |
| 2008 | Respondent driven sampling | 204  81 | Johannesburg  Durban | 49.5% (42.5-56.5)  27.5% (17.0-38.1) | (Rispel, Metcalf et al. 2011b) |
| 2009 | Venue-based with peer-referral at each venue | 200 | Cape Town | 25.5% (19.6-32.1) | (Baral, Burrell et al. 2011) |
| 2010 | Outreach workers and through peer-referral. | 316 | Cape Town | Not available | (Tucker, Liht et al. 2013, Tucker, Liht et al. 2014) |
| 2012  2012/13  2012/13 | Respondent driven sampling | 286  290  349 | Cape Town  Durban  Johannesburg | 22.3% (14.7–30.1)  48.2% (37.9–55.4)  26.8% (20.4–35.6) | (Cloete, Jooste et al. 2014) |
| 2012 | MSM attending a men’s Health clinic | 200 | Cape Town | 44.0% (37.0-51.2) | (Rebe, Lewis et al. 2015, Muller, Rebe et al. 2016) |
| 2012 | Previously-developed lists of MSM and snowball sampling. | 34 | Cape Town and Port Elizabeth | Not available | (Siegler, Voux et al. 2014) |
| 2015 | Event- and venue-based, online, participant referral, and walk-ins at study clinics. | 115  177 | Cape Town  Port Elizabeth | 30.4% (22.7-39.5)  50.8% (43.5-58.2) | (McNaghten, Kearns et al. 2014) |
| Overall female | | | | | |
| 2002 | Multi-stage stratified cluster sampling | 4656 | National | 17.7% (15.2-20.4) | (Human Sciences Research Council) |
| 2005 | Multi-stage stratified cluster sampling | 6547 | National | 20.2% (18.3-22.2) | (Human Sciences Research Council) |
| 2008 | Multi-stage stratified cluster sampling | 8327 | National | Not available | (Human Sciences Research Council) |
| 2012 | Multi-stage stratified cluster sampling | 9982 | National | 23.2% (21.3-25.1) | (Human Sciences Research Council) |
| 2017 | Multi-stage stratified cluster sampling | 12,900 aged 15-64 | National | 26.3% (24.5-28.2) | (Human Sciences Research Council) |
| Overall male | | | | | |
| 2002 | Multi-stage stratified cluster sampling | 3772 | National | 12.80% | (Human Sciences Research Council) |
| 2005 | Multi-stage stratified cluster sampling | 4078 | National | 11.7% (10.0-13.6) | (Human Sciences Research Council) |
| 2008 | Multi-stage stratified cluster sampling | 5501 | National | Not available | (Human Sciences Research Council) |
| 2012 | Multi-stage stratified cluster sampling | 7561 | National | 14.5% (12.8-16.3) | (Human Sciences Research Council) |
| 2017 | Multi-stage stratified cluster sampling | 9667 aged 15-64 | National | 14.8% (13.3-16.5) | (Human Sciences Research Council) |

## Supplementary Table 2. Demographic, sexual and behavioural parameters of female sex workers (FSWs) and their clients.

| **Parameter definition** | **Parameter Symbol** | **Clients** | **FSW** | **References** |
| --- | --- | --- | --- | --- |
| Proportion of adults that are clients or FSW |  | To balance commercial sex acts | 0.69-0.96% | (SWPSSES 2013): A sex worker size estimation study estimated 0.69-0.96% of adult females are female sex workers. We do not have client population size estimates except from 2005/08 general population HSRC surveys(Human Sciences Research Council , Human Sciences Research Council) which showed that <2% of adult men reported having commercial partners, which will be an under estimate. In their modelling of SA, Bekker and Johnson assumed a high estimate of 35%(Bekker, Johnson et al. 2015). In a review of size estimate studies of males who report having paid sex, Carael found the median estimate of the proportion of men in Southern Africa who paid to have sex in the last 12 months to be 7.0% (IQR: 1.7-10.6%) (Carael, Slaymaker et al. 2006). Another survey found that that 6.4% (95%CI: 3.5-10.8%) of men reported having ever paid for sex(Quaife, Eakle et al. 2016). However, in light of the limited data, and its possible uncertainty and biases we calibrate the client population size to balance the number of commercial sex acts that FSW and clients report. We impose an upper bound of 35% to the proportion of men that are clients based on the assumption by Bekker and a high estimate of ~30% in Benin(Behanzin, Diabate et al. 2013). |
| Frequency of FSW per client and client per FSW per year | $n_{3}^{co}$, $n_{4}^{co}$ | 2.3-72.8 per year | 100-1000 per year | (SAHMS-FSW 2014) reported a min and max range of 50-200 paying clients in last 6 months across 3 cities. (Quaife, Eakle et al. 2016) reported a mean of 35.9 (27.3-44.6) and median of 20 (IQR 10-40 across 3 age groups) clients in previous week. A survey in Port Elisabeth among FSW (PEFSW 2015) had a mean of 5.4 (95%CI: 4.8-6.2) and median of 4 (IQR 0-7) new clients in the last 30 days and mean of 6.9 (95%CI 6.3-7.6) and median 5 (IQR: 3-9) regular clients in last 30 days. The same study reported a mean of 3.8 (2.7-4.9) clients per day with a median of 15 (12-20) days of sex work per month. We assume a lower bound from the SAHMS study as it was a combination of FSW in 3 different cities. Upper bound is a compromise between studies that have reported very high frequency and those with low frequency of clients. We also consider that regular FSW also take time off sex work and therefore have frequency of clients per FSW of (100-1000).  In terms of FSWs seen by clients, the HSRC surveys (Human Sciences Research Council) and (Human Sciences Research Council) report 2.3 and 3.4 commercial partners in the last year among males who report having them in last 12 months which seems low. Conversely, in the Port Elisabeth client survey, clients report a median of 2 (IQR: 1-4) and mean of 3.5 (95%CI: 3.3-4.3) commercial sex acts in the past 3 months. In the Klerksdorp client survey, clients reported a median of 3 (IQR: 2-4) and an average of 3.4 (95%CI: 2.8-3.9) commercial sex acts in the past month and a median of 1 (IQR: 1-1) and average of 1.3 (95%CI: 1.1-1.4) commercial sex acts in the past week. We assume lower bound from the HSRC data and upper bound from the Klerksdorp survey. |
| Percentage of commercial sex acts that are anal | $p_{3}^{co}$, $p_{4}^{co}$ | 0.6-9.3% | 0.6-9.3% | On average, in the Port Elisabeth surveys, FSW report 2.2% (95%CI 1.1-3.3%) of commercial sex acts are anal (PEFSW 2015, Rao, Baral et al. 2016). On average, clients report 3.9% (95%CI: 3.0-4.9) of commercial sex acts are anal. We assumed the amount of AI by Clients and FSW was the same and took the lower bound from what FSWs report and upper bound from what clients report to produce a range. We incorporate additional uncertainty by halving and doubling the odds associated with the lower and upper values in the range. |
| Condom use VI with commercial partners | $\pi_{3v}^{co}$, $\pi_{4v}^{co}$ | Time varying | Time varying | See separate section on condom use trends |
| Condom use for AI with commercial partners | $\pi_{3v}^{co}$, $\pi_{4v}^{co}$ | Time varying | Time varying | See separate section on condom use trends |
|  |  |  |  |  |
| Proportion of clients and FSW that have a main partner in last 12 months | $p_{3}^{m}$, $p_{4}^{m}$ | 0.81-0.98 | 0.25-0.90 | In the Ekurhuleni survey, 0.85 (0.80-0.90) of FSW reported having any main partners (Quaife, Eakle et al. 2016). In contrast in the (SAHMS-FSW 2014), 0.25-0.52 of FSW had main partners in the last 6 months. Lastly, in the PE survey, 0.66 (0.61-0.70) of FSW reported having a long-term partner in the last 12 months (PEFSW 2015, Rao, Baral et al. 2016). For FSWs, we assumed the minimum and maximum values from these FSW surveys.  In the PE client survey, 84.5% (95%CI: 81.2-87.3) of clients report having a main female partner in the last 12 months. In the Klerksdorp client survey, 94.3% (95%CI: 88.1-97.9%) of clients had a main partner in the last 12 months. For clients, we assumed the minimum and maximum from these surveys. |
|  |  |  |  |  |
| Frequency of main partners for clients and FSW among those with main partners (per year) | $n_{3}^{m}$, $n_{4}^{m}$ | 1.0-2.9 per year | 1.0-3.07 per year | (SAHMS-FSW 2014) report a median of 1 and mean of 1.02-3.07 (95%CI range across settings) main partners for FSW in the previous 6 months. Among those with commercial partners, females had an average of 1.2 main partners and males 1.3 in the 2005 HSRC survey (Human Sciences Research Council), while in 2008 females report 0.68 and males had reported 2.7 in the last 12 months (Human Sciences Research Council). In the PE survey, the frequency of main partners among FSW who report sex with non-paying partners in the last 30 days was 0.91 (0.24-1.58) and among those who report long term partners in the last 12 months was 1.12 (0.68-1.7) we took the upper and lower bounds from these estimates for FSWs  In Port Elisabeth, clients with at least one main partner report a median of 1 (IQR: 1-2) and average of 2.45 (95%CI: 2.05-2.86) main partners in the past 12 months. In Klerksdorp, clients with at least one main partner report a median of 1 (IQR: 1-1) and average of 1.4 (95%CI: 1.1-1.6) main partners in the past 12 months. We took the upper and lower bounds from these estimates for clients. |
| Frequency of vaginal sex with main partners among those reporting main partners for clients and FSW per year | $\psi_{3v}^{m},\psi_{4v}^{m}$ | 6-144 per year | 24-144 per year | In the 3 city FSW survey, 15-60 (95%CI across cities) or 8-90 (IQR across cities) vaginal sex acts in the last 6 months were reported by FSW with their main partners (SAHMS-FSW 2014). In the Port Elisabeth survey, the number of vaginal sex acts with long term partners among those who have them was reported as 8 (2.0-12.0) in the last 30 days (PEFSW 2015, Rao, Baral et al. 2016), while number of sex acts was 4.0 (2.9-5.0) in the previous month in the Ekurhuleni FSW survey (Quaife, Eakle et al. 2016). Assume minimum and maximum from these FSW surveys including the 95%CI from (SAHMS-FSW 2014).  In Port Elisabeth, clients report a median of 3 (IQR: 1-6) and an average of 5.2 (95%CI: 4.6-5.9) vaginal sex acts with main partners in the past 30 days. For clients, we use the IQR from PE and we incorporate additional uncertainty by halving and doubling the lower and upper bounds. |
| Frequency for AI with main partners for clients and FSW per year | $\psi_{3a}^{m},\psi_{4a}^{m}$ | 0-7.2 per year | 1.6-60.0 per year | In the 3 city FSW survey, FSW reported 1.0-36.0 (IQR across cities) or 2.0-30.0 (95%CI across cities) anal sex with main partners in last 6 months (SAHMS-FSW 2014). In the Port Elisabeth FSW survey, FSWs reported 1.3 (0.13-2.45) anal sex acts with their long term partners in last 30 days (PEFSW 2015, Rao, Baral et al. 2016) whereas FSW in the Ekurhuleni survey reported a mean of 0.35 AI acts with regular partners in the last month (Quaife, Eakle et al. 2016). Consider range of frequency of anal sex as minimum and maximum from these studies.  In Port Elisabeth, clients report a median of 0 (IQR: 0-0) and an average of 0.17 (95%CI: 0.02-0.3) anal sex acts with main partners in the past 30 days. For clients, we consider lower bound for clients from the lower IQR and upper bound from the 95% CI and we incorporate additional uncertainty by halving and doubling the lower and upper bounds. |
| Condom use VI with main partners for clients and FSW | $\pi_{3v}^{m}$, $\pi_{4v}^{m}$ | Time varying | Time varying | See section on condom use trends |
| Condom use AI with main partners for clients and FSW | $\pi_{3a}^{m}$, $\pi_{4a}^{m}$ | Time varying | Time varying | See section on condom use trends |
| Proportion of clients and FSW with casual partners | ${p_{3}^{c},p}_{4}^{c}$ | 0.534-0.979 | 0.056-0.29 | In the 3 city FSW survey (SAHMS-FSW 2014), 5.6-29.2% of FSW reported casual partners in the last 6 months. In the Port Elisabeth FSW survey, 12-19% of FSW had casual partners in the last year (PEFSW 2015, Rao, Baral et al. 2016), while in the Ekurhuleni FSW survey, 67.0% (95%CI: 60.5-73.5) of FSW reported having casual partners in the previous year (Quaife, Eakle et al. 2016). We did not use data from the Ekurhuleni survey because it was not consistent with other surveys, and so use the minimum and maximum from the other surveys.  In the Port Elisabeth client survey, 57.6% (95%CI: 53.4-61.7) of clients reported having a casual female partner in the last 3 months. In the Klerksdorp client survey, 94.4% (95%CI: 88.3-97.9) of clients reported having a casual partner in the past year. We took the upper and lower bounds from these estimates for clients. |
| Frequency of casual partners for clients and FSW per year among those with casual partners | ${n_{3}^{c},n}_{4}^{c}$ | 1.1-15.1 per year | 1.0-18.0 per year | In the 3 city FSW survey (SAHMS-FSW 2014), FSW had 1-5 (range of 95% CI across cities) or 1-9 (for range of IQR across cities) casual partners in the previous 6 months. In the Port Elisabeth FSW survey, FSW (PEFSW 2015, Rao, Baral et al. 2016) had a mean of 1.38 (0.34-2.41) casual partners in the past year. Lastly, In the HSRC 2005 survey, females report 0.85 non-regular partners among those with commercial partners in last 12 months (Human Sciences Research Council). We assume the range from these estimates.  In the HSRC 2005 survey, men report an average of 1.1 non-regular partners among those with commercial partners in last 12 months (Human Sciences Research Council). Conversely, in the Port Elisabeth client survey, Clients report a median of 2 (IQR: 1-3) and average of 3.0 (95%CI: 2.6-3.4) casual partners in the past 3 months. In the Klerksdorp survey, Clients report a median of 8 (IQR: 5-11) and average of 12.0 (95%CI: 8.8-15.1) casual partners in the past year. We assume the range from these estimates. |
| Frequency of VI acts for clients and FSWs with casual partners among those with casual partners per month | $\psi_{3v}^{c},\psi_{4v}^{c}$ | 0.5-10.2 per month | 0.2-8.3 per month | In the 3 city FSW survey (SAHMS-FSW 2014), FSW reported 1-33 (range of 95%CI across 3 cities, 1-50 if range of IQR) VI with casual partners in the last 6 months, whereas the port Elisabeth survey reported a median of 3 (1.5-4.5) in the last 30 days (PEFSW 2015, Rao, Baral et al. 2016). We used the range in the monthly estimates to give a sampling range.  In the Port Elisabeth client survey, clients report a median of 2 (IQR: 1-4) and a mean of 4.2 (95%CI: 3.5-5.1) vaginal sex acts with casual partners in the past 30 days. Because the data is skewed, we consider lower bound for clients from the lower IQR and upper bound from the 95% CI in the PE clients survey and we incorporate additional uncertainty by halving and doubling the lower and upper bounds. |
| Frequency of AI acts for clients and FSWs with casual partners among those with casual partners per month | $\psi_{3a}^{c},\psi_{4a}^{c}$ | 0.0-0.52 per month | 0.0-3.3 per month | In the 3 city FSW survey (SAHMS-FSW 2014), FSW reported 1.0-17.0 (IQR across cities) or 1.75-20.0 (95%CI across cities) AI sex acts with casual partners in the last 6 months, while another FSW survey from Ekurhuleni reported a mean of 0-5.6 acts in the last year (Quaife, Eakle et al. 2016). FSW in the port Elisabeth survey reported no AI with casual partners. We used the range of the monthly estimates to give a sampling range.  In Port Elisabeth, clients report a median of 0 (IQR: 0-0) and an average of 0.1 (95%CI: 0.04-0.2) anal sex acts with casual partners in the past 30 days. We consider the lower bound for clients from the lower IQR (0) and upper bound from the range assumed for low risk males (0.26). We incorporate additional uncertainty by halving and doubling the lower and upper bounds. |
| Condom use VI with casual partners for clients and FSWs | $\pi_{3v}^{c}$, $\pi_{4v}^{c}$ | Time varying | Time varying | See separate section |
| Condom use AI with casual partners for clients and FSWs | $\pi_{3a}^{c}$, $\pi_{4a}^{c}$ | Time varying | Time varying | See separate section |
|  |  |  |  |  |
| Duration of buying sex and sex work for clients and FSWs in years | $1/g,1/\gamma$ | 1.5-36 years | 3.2-8.1 years | In the 3 city FSW survey (SAHMS-FSW 2014), FSW reported a duration of sex work IQR of 3-10 years with a mean of 7.3 years, while the port Elisabeth FSW survey (PEFSW 2015, Rao, Baral et al. 2016) reported a mean duration of 5.4 (2.4-8.4) years and median of 4 years with IQR of (2.0-7.0). In the Ekurhuleni FSW survey, the min and max from the IQR for the duration of sex work of the 3 age groups is 0-6 years (Quaife, Eakle et al. 2016).  We used a simple model of FSW initiation, aging and cessation to estimate cessation rates using data from SAHMS. This data gives a range of possible cessation rates of 3.2-8.1 years, agreeing with data from Port Elizabeth and Ekurhuleni.  In the Port Elisabeth client survey, clients report a median of 11.5 (IQR: 6-18) years and mean of 12.9 (95%CI: 12.0-13.7) years duration of buying sex. In the Klerksdorp client survey, clients report a median of 5 (IQR: 3-8.5) years and mean of 6.4 years (95%CI: 5.4-7.4) of buying sex. We multiply the lower bound by 0.5 and upper bound by 2 because of uncertainty in how overall duration of sex work relates to current duration of sex work. |
| Relative difference in ART coverage between clients and all males and FSWs and all females. |  | 0.7-1.0 | 0.7-1.0 | In the port Elisabeth FSW survey, 102 FSW reported that they were currently on ARVs (PEFSW 2015), out of 261 FSW that tested HIV+ (39.1% (33.1-45.3%) coverage). Among self-disclosed HIV positive FSW from the 3 city FSW survey, 373 reported current ART uptake, giving an ART coverage of 29.6% (27.1-32.2%) among HIV+ FSWs (SAHMS-FSW 2014). In the Ekurhuleni FSW survey, out of 81 FSW who reported that they were HIV+, 48 reported currently being on ART (Quaife, Eakle et al. 2016), giving ART coverage of 59.3% (47.8-70.0%). In the Soweto FSW survey, 118 out of 269 (43.9%; 95%CI: 37.8-50.0) FSW testing HIV positive self-reported current treatment, with 99 (70%) of those with viral loads less than 400 copies/ml (Coetzee, Hunt et al. 2017). We compare this with UNAIDs estimates for females [2013: 42 (31-51); 2014: 48 (36-56); 2015: 53 (40-65); 2016: 59 (45-73)] and assume ART coverage among FSW is 0.7-1.0 times that among all females.  For clients, the Port Elisabeth survey (2017-18) found 31/83 (37.3%, 95%CI: 27.5-48.4) of HIV positive clients reported being on ART and 29.2% (95%CI: 19.7-40.4%) of HIV positive clients were virally supressed (< 1000 copies/ml). In the Klerksdorp survey, 32.3% (95%CI: 16.8-51.4%) of HIV positive clients reported being on ART and 8/30 (26.7%, 95%CI: 9.9-42.3) of clients were virally supressed (<1000 copies/ml).  UNAIDs coverage among all males is 53% (38-66%) in 2017 so assume ART coverage among males is 0.7-1 times that among all males. |
| Proportion of male adults that are circumcised. | $\zeta$ | Time varying | Not applicable. | See section on circumcision trends. |

## Supplementary Table 3. Demographic, sexual and behavioural parameters of low risk males (LRM) and females (LRF).

| **Parameter definition** | **Parameter Symbol** | **Low risk male** | **Low risk female** | **References** |
| --- | --- | --- | --- | --- |
| Proportion of LRF and LRM with main partner | $p_{1}^{m},p_{2}^{m}$ | 0.75-0.83 | 0.71-0.77 | Proportion of males and females with regular partners are (0.75-0.83) and (0.71-0.77) from HSRC general population surveys in 2005 and 2008 (Human Sciences Research Council), (Human Sciences Research Council). Conversely, in the survey from Ekurhuleni, 0.92 (0.88-0.96) of males and 0.88 (0.84-0.93) of females reported having any regular partners (Quaife, Eakle et al. 2016). Just use HSRC data because more representative than the Ekurhuleni survey. |
| Frequency of main partners among those with main partners for LRF and LRM per year | $n_{1}^{m},n_{2}^{m}$ | 1.10-2.8 per year | 1.0-1.3 per year | Male and female report 1.12 (1.06-1.22) and 1.01 (1.00-1.02) regular partners in the last 12 months, respectively, in the HSRC surveys (Human Sciences Research Council), (Human Sciences Research Council). Conversely, in the Ekurhuleni survey, the number of regular partners reported by male and female in the previous year was 2.1 (1.4-2.8) and 1.2 (1.1-1.3), respectively (Quaife, Eakle et al. 2016). Used range including Quaife and HSRC data. |
| Frequency of VI sex with main partners for LRM and LRF partners per year | $\psi_{1v}^{m},\psi_{2v}^{m}$ | 42.0-70.8 per year | 42.0-70.8 per year | Frequency of sex in last 30 days among those with regular partners was (4.6-5.9) for male and (3.7-4.6) for female in the 2005 HSRC survey (Human Sciences Research Council). Conversely, in the Ekurhuleni study (Quaife, Eakle et al. 2016), males and females reported 3.8-5.7 and 3.5-5.1 sex acts in the previous month, respectively. Assume (3.5-5.9) per month for both |
| Frequency of AI sex with main partners for LRM and LRF per year | $\psi_{1a}^{m},\psi_{2a}^{m}$ | 1.2-13.2 per year | 1.2-13.2 per year | In the Ekurhuleni survey, males and females report a mean of 0.93-0.99 and 0.96-1.0 anal acts in last month with regular partners (Quaife, Eakle et al. 2016). There was no data on this from the HSRC study.  A systematic review of heterosexual anal sex in South Africa found that across 7 studies frequency of anal sex varied between 0.1-1.1 per month (Owen, Elmes et al. 2017). Only one study reported frequency of anal sex by partnership type - a 2003 study in Soweto of HIV negative males and females. Study participants reported an average of 0.4 and 0.1 anal sex acts with steady partners and casual partners in the past 6 months, respectively. (Andersson, Van Niekerk et al. 2009). We use the range found in the systematic review for both males and females, as this range includes estimates from the Ekurhuleni study, which suggests similar frequency of anal sex with main partners by gender. |
| Condom use for VI with main partner for LRM and LRF | $\pi_{3v}^{m}$, $\pi_{4v}^{m}$ | Time varying | Time varying | See separate section |
| Condom use AI with main partner | $\pi_{3a}^{m}$, $\pi_{4a}^{m}$ | Time varying | Time varying | See separate section |
| Proportion of LRM and LRF with casual partners | $p_{1}^{c},p_{2}^{c}$ | 0.08-0.49 | 0.02-0.27 | In the HSRC surveys, a proportion of 1.6-3.0% for female and 8.0-9.0% for males had non-regular partners in last 12 months (Human Sciences Research Council), (Human Sciences Research Council). Conversely, the Ekurhuleni survey found that 0.43 (0.36-0.49) of males and 0.21 (0.16-0.27) of females reported having any casual partners in the previous year (Quaife, Eakle et al. 2016) . Assume size of group with casual partners is larger than that reported in 2005/08 HSRC. Assume lower bound reported in (Human Sciences Research Council), (Human Sciences Research Council) and upper bound as reported from (Quaife, Eakle et al. 2016). |
| Frequency of casual partners for LRM and LRF per year | $n_{1}^{c},n_{2}^{c}$ | 1.7-2.9 per year | 1.7-2.9 per year | Data direct from HSRC surveys for those that report these non-regular partnerships (Human Sciences Research Council), (Human Sciences Research Council) |
| Frequency of VI sex with casual partners for LRF and LRM per month | $\psi_{1v}^{c},\psi_{2v}^{c}$ | 1.1-4.3 per month | 1.1-4.3 per month | In the Ekurhuleni survey, males and females report 2.1-4.3 and 1.1-4.1 sex acts in previous month with casual partners among those reporting casual partners (Quaife, Eakle et al. 2016). Data from old RHRU survey in 2000 shows 1.5-3.0 sex acts with casual partners. No data on this from the HSRC surveys. Use lower and upper bounds. |
| Frequency of AI sex with casual partners for LRF and LRM per month | $\psi_{1a}^{c},\psi_{2a}^{c}$ | 0.02-0.26 per month | 0-0.22 per month | In the Ekurhuleni survey, males and females report a mean of 0.75-3.1 and 0-0.74 anal sex acts with casual partners in the last year, respectively (Quaife, Eakle et al. 2016).  A systematic review of heterosexual anal sex in South Africa found that across 7 studies frequency of anal sex varied between 0.1-1.1 per month (Owen, Elmes et al. 2017). Only one study reported frequency of anal sex by partnership type - a 2003 study in Soweto of HIV negative males and females. Study participants reported an average of 0.4 and 0.1 anal sex acts with steady partners and casual partners in the past 6 months, respectively. (Andersson, Van Niekerk et al. 2009). Based on the Soweto and Ekurhuleni studies, we assume frequency of anal sex with casual partners is at most ¼ of that amongst main partners. Applying this to the range found in the systematic review gives 0.02-0.22. We assume this range applies to both males and females as a Cape Town study found similar levels of frequency of anal sex among young men and women (Simbayi, Kalichman et al. 2005). We use the minimum and maximum from the Ekurhuleni study and the adjusted range in the systematic review. |
| Condom use VI with casual partners for LRM and LRF | $\pi_{3v}^{c}$, $\pi_{4v}^{c}$ | Time varying | Time varying | See separate section |
| Condom use with casual partner for AI | $\pi_{3a}^{c}$, $\pi_{4a}^{c}$ | Time varying | Time varying | See separate section |
| ART coverage among adult male and female who are HIV+ |  | Time varying | Time varying | ART coverage trends from UNAIDS (UNAIDS-AIDSinfo) |
| Inflow of HIV positive into the adult population | $\phi_{1},\phi_{2}$ | Time varying | Time varying | Data from the HSRC studies suggest the proportion of 15-year old HIV positive females who enter the adult population is 4.9% (0.98-21.6%) in 2002, 7.5% (3.7-14.7%) in 2005 and 3.7% (1.6-8.4%) in 2008. The corresponding proportion of HIV positive 15-year-old males is 6.7% (1.6-24.4%), 1.6% (0.56-4.8%) and 1.5% (0.38-6.8%), respectively (Human Sciences Research Council , Human Sciences Research Council , Human Sciences Research Council). We assume the inflow of HIV positive male and female increase linearly from 0 in 1995 to the values in 2002, 2005, 2008 and stay constant thereafter. |
| Proportion of male adults that are circumcised. | $\zeta$ | Time varying | Not applicable. | See section on circumcision trends. |

For all the SAHMS estimates where both 95%CI and IQR across the three sites are given, we used the 95%CI range.

## Supplementary Table 4. Demographic, sexual and behavioural parameters of men who have sex with men (MSM).

| **Parameter definition** | **Parameter Symbol** | **Male partners** | **Female partners** | **References** |
| --- | --- | --- | --- | --- |
| Proportion of male adults that are MSM |  | 0.65-7.3% | | Cross-sectional household survey in 2008 (Dunkle, Jewkes et al. 2013) – in 3 adjoining districts of the Eastern Cape and KwaZulu-Natal provinces of South Africa found 4.2% (95%CI: 3.3-5.3) reported lifetime consensual sex (oral, anal or unspecified). Baseline questionnaire in 2002-3 for RCT amongst sexually experienced Xhosa males (Jewkes, Dunkle et al. 2006) found 3.6% (95%CI: 2.6-4.8) had had sexual contact with a man (~3/4 of whom report having only one such event which was coerced). 3.2% of men who participated in the 2008 HSRC household survey self-reported same-sex behaviour (Human Sciences Research Council). 176/2769 (6.4% 95%CI: 5.5-7.3%) men surveyed in urban townships and a STI clinic in Cape Town reported same sex partners in the last 3 months, of which 51% had engaged in anal intercourse (Kalichman, Simbayi et al. 2009). |
| Age at which MSM first have sex with a man |  | 15-19 | | 2008 study in Johannesburg and Durban (Rispel, Metcalf et al. 2011b) reports a median age at first sex with a man of 17.0 years, range: 5-32; mean: 16.7. |
| Proportion of MSM that have a main partner in last 12 months | $p_{5}^{m,m}$, $p_{6}^{m,m}$,  $p_{5}^{m,f}$, $p_{6}^{m,f}$ | 46.0-77.5% | 2.8-40.6% | 8% (16/200) of MSM in Cape Town reported having a regular female partner in 2009 (Baral, Burrell et al. 2011). In the South Africa Marang Men’s project (2012-13), 2.8%, 16.0% and 38.7% of MSM reported having a regular female partner in Durban, Cape Town and Johannesburg, respectively (Cloete, Jooste et al. 2014). In the Mpumalanga Men’s study (2012-2013), 25.7% (95%CI: 18.05-33.2) and 40.6% (95% CI 32.2-48.9) of MSM in Gert Sibande and Ehlanzeni, respectively, reported having a regular female partner (Lane, Osmand et al. 2014). In the Soweto Men’s Project (2008), 51.2% (RDS adjusted: 63.4%, 95%CI: 55.9-70.8) of MSM reported having a regular female partner (Lane, Raymond et al. 2011). In the HIVST study (2015), 69.1% and 37.5% of **HIV negative** MSM reported having a regular female partner in Gert Sibande and Ehlanzeni, respectively (Lippman, Lane et al. 2018). A 2012 cross-sectional survey of MSM attending a MSM sexual health care clinic found that 22.5% of MSM reported having any female sexual partners in the last 12 months (Rebe, Lewis et al. 2015). In the Sibanye Health Project (2015), 14.5% (95%CI: 10.0 – 20.7) and 15.3% (95%CI: 9.7 – 23.3) of MSM in Port Elizabeth and Cape town, respectively, report having a regular female partner. In the Johannesburg/eThekwini Men’s Study (2008), 64.2% (95%CI: 58.3-69.8) of MSM in Johannesburg and Durban reported never having sex with a woman (Rispel, Metcalf et al. 2011b). We do not use the HIVST study, as this is a sample of HIV negative MSM only, or the Soweto Men’s project, as this is inconsistent with other data. We use the minimum and maximum from the other surveys.  In the Johannesburg/eThekwini Men’s Study (2008), 62.4% (95%CI: 56.6-68.1) of MSM in Johannesburg and Durban reported having a male main partner (Rispel, Metcalf et al. 2011b). In 2008, 55.4% (95%CI: 49.6-61.2) of MSM in Pretoria reported being in a steady relationship with another man (Knox, Yi et al. 2010). In the Soweto Men’s Project (2008), 73.0% (RDS adjusted: 69.6%, 95%CI: 64.7-76.7) of MSM reported having a regular male partner (Lane, Raymond et al. 2011). 46% (95%CI: 41.8-56.1) of MSM in Cape Town reported having a regular male partner in 2009 (Baral, Burrell et al. 2011). A 2012 cross-sectional survey of MSM attending a MSM sexual health care clinic found that 52% (95% CI: 44.8-59.1%) of MSM reported having a male partner who was their primary sexual partner (Rebe, Lewis et al. 2015). In the South Africa Marang Men’s project (2012-13), 64.8%, 62.8% and 62.9% of MSM reported having a regular male partner in Durban, Cape Town and Johannesburg, respectively (Cloete, Jooste et al. 2014). In the Mpumalanga Men’s study (2012-2013), 66.0% (95% CI: 58.2-73.7) and 74.2% (95%CI: 65.9-79.9) of MSM in Gert Sibande and Ehlanzeni, respectively, reported having a regular male partner (Lane, Osmand et al. 2014).In the HIVST study (2015), 80.0% and 86.1% of HIV negative MSM reported having a regular male partner in Gert Sibande and Ehlanzeni, respectively (Lippman, Lane et al. 2018). In the Sibanye Health Project (2015), 73.6 (95%CI: 66.2-79.9) and 77.5% (95%CI: 68.2-85%) of MSM in Port Elizabeth and Cape town, respectively, report having a regular male partner.  We do not use the HIVST study, as this is a sample of HIV negative MSM only, and use the minimum and maximum from the other surveys. |
|  |  |  |  |  |
| Frequency of main partners per year for young MSM among those with main partners in the past 12 months | $n_{5}^{m,m}$, $n_{6}^{m,m}$,  $n_{5}^{m,f}$, $n_{6}^{m,f}$ | 1-2.01 per year | 1-2.68 per year | In the Sibanye Health Project (2015), young MSM with main male partners reported a median of 1 (IQR 1:2) in both Cape Town (mean: 1.79, 95%CI 1.57-2.01) and Port Elizabeth (mean: 1.47, 95%CI 1.35-1.59) male main partners in the last 12 months. Use median as lower bound and the maximum of the upper 95% confidence limits as the upper bound.  In the Sibanye Health Project (2015), young MSM with main female partners reported a median of 1 (IQR 1:2) and 1 (IQR 1-1) female main partners in the last 12 months in Cape Town (mean: 2.0, 95%CI 1.32-2.68) and Port Elizabeth (mean: 1.23, 95%CI 1.08-1.39), respectively. Use median as lower bound and the maximum of the upper 95% confidence limits as the upper bound. |
| Relative increase in frequency of main partners if age>30 vs age<30. |  | 1.09-1.54 | 0.44-0.94 | In the Sibanye Health Project (2015), amongst men with at least 1 main male partner in the past 12 months, older MSM have (aIRR adjusted for site) 1.30 (95%CI: 1.09-1.54) times more main male partners than young MSM.  In the Sibanye Health Project (2015), amongst men with at least 1 main female partner in the past 12 months, young MSM had 0.65 (95%CI: 0.44-0.94) times less main female partners than older MSM. |
| Frequency of vaginal sex with main partners per year among those reporting main partners | $\psi_{5v}^{m},\psi_{6v}^{m}$ | Not applicable | 12-120 per year | In the Sibanye Health Project (2015), MSM in Cape Town and Port Elizabeth reported a median of 7.25 (IQR 3-30) vaginal sex acts with female main partners in the last 3 months (mean: 16.4, 95%CI: 10.3-22.4). |
| Frequency of AI per year with main partners among those reporting main partners. | $\psi_{5a}^{m,m},\psi_{6a}^{m,m}$,  $\psi_{5a}^{m,f},\psi_{6a}^{m,f}$ | 12-120 per year | 0-28 per year | In the Sibanye Health Project (2015), MSM in Cape Town reported a median of 6 (IQR 3-10) anal sex acts with male main partners in the last 3 months (mean: 8.9, 95%CI: 6.5-11.2); MSM in Port Elizabeth reported a median of 6 (IQR 3:30) anal sex acts with male main partners in the last 3 months (mean: 13.9, 95%CI 11.1-16.7).  In the Sibanye Health Project (2015), MSM in Cape Town and Port Elizabeth reported a median of 3 (IQR 0-7) anal sex acts with female main partners in the last 3 months (mean: 6.7, 95%CI: 1.9-11.5). |
| Condom use VI with main partners | $\pi_{5v}^{m}$, $\pi_{6v}^{m}$ | Not applicable | Time varying | See section on condom use trends |
| Condom use AI with main partners. | $\pi_{5a}^{m,m}$, $\pi_{6a}^{m,m}$,  $\pi_{5a}^{m,f}$, $\pi_{6a}^{m,f}$ | Time varying | Time varying | See section on condom use trends |
| Proportion of MSM with casual partners | ${p_{5}^{c,m},p}_{6}^{c,m}$  ${p_{5}^{c,f},p}_{6}^{c,f}$ | 60.1-74.4% | 6.5-17.0% | In the Sibanye Health Project (2015), 73.7% (95%CI: 64.1-81.5) and 67.7% (95%CI: 60.1-74.4) of all MSM in Cape town and Port Elizabeth, respectively, report having a casual male partner.  In the Sibanye Health Project (2015), 9.6% (95%CI: 6.5 – 14.0) and 13.3% (95%CI: 10.3- 17.0) of young MSM in Cape town and Port Elizabeth, respectively, report having a casual female partner. |
| Increase in the odds of having a casual partner if older (>30) vs young (<30) |  | - | 1.02-2.42 | In the Sibanye Health Project (2015), older MSM have 1.57 higher odds (95%CI: 1.02-2.42) of having a casual female partner.  There was no difference between the proportion of young (74.3%; 95%CI: 68.1-79.7) and older (72.0% 95%CI: 60.6-81.2) MSM who report having a casual male partner. |
| Frequency of casual partners per year among Young MSM with casual partners | $n_{5}^{c}$ | 1-4 per year | 1-6.5 per year | In the Sibanye Health Project (2015), young MSM reported a median of 2 (IQR 1-3) and 2 (IQR: 1-4) male casual partners in the last 12 months in Cape Town (mean: 3.2, 95%CI: 2.6-3.8) and Port Elizabeth (mean: 2.74, 95%CI: 2.39-3.08), respectively.  In the Sibanye Health Project (2015), young MSM reported a median of 2 (IQR 1-2) and 2 (IQR: 1-6.5) female casual partners in the last 12 months in Cape Town (mean: 3.9, 95%CI: 1.2-6.5) and Port Elizabeth (mean: 3.8, 95%CI: 1.3-6.2), respectively. |
| Relative increase in frequency of casual partners if age>30 vs age<30. |  | 1.12-1.59 | 1.06-2.21 | In the Sibanye Health Project (2015), amongst men with at least 1 male casual partner in the past 12 months, older MSM have (aIRR, adjusted for site) 1.34 (95%CI: 1.12-1.59) times more casual male partners than young MSM.  In the Sibanye Health Project (2015), amongst men with at least 1 female casual partner in the past 12 months, older MSM have (aIRR, adjusted for site) 1.53 (95%CI: 1.06-2.21) times more casual female partners than young MSM. |
| Frequency of VI acts with casual partners among those with casual partners per partner | $\psi_{3v}^{c},\psi_{4v}^{c}$ | Not applicable | 2-6.5 | In the Sibanye Health Project (2015), MSM in Cape Town and Port Elizabeth reported a median of 3.5 (IQR 2-6.5) vaginal sex acts with female casual partners in the last 3 months (mean: 3.9, 95%CI: 2.4-5.4). |
| Frequency of AI acts with casual partners among those with casual partners per month | $\psi_{3a}^{c},\psi_{4a}^{c}$ | 2-10.7 per month | 0-5 per month | In the Sibanye Health Project (2015), MSM in Cape Town reported a median of 3 (IQR 2:6) anal sex acts per male casual partners in the last 3 months (mean: 4.2, 95%CI: 3.2-5.2); MSM in Port Elizabeth reported a median of 3 (IQR 2:6) anal sex acts per male casual partners in the last 3 months (mean: 7.9, 95%CI 5.1-10.7).  In the Sibanye Health Project (2015), MSM in Cape Town and Port Elizabeth reported a median of 2 (IQR 0-5) anal sex acts per female casual partners in the last 3 months (mean: 2.5, 95%CI: 1.0-4.1).  We assume that all sex acts with casual partners occurred over 1 month. |
| Condom use VI with casual partners. | $\pi_{3v}^{c}$, $\pi_{4v}^{c}$ | Time varying | Time varying | See separate section |
| Condom use AI with casual partners | $\pi_{3a}^{c}$, $\pi_{4a}^{c}$ | Time varying | Time varying | See separate section |
| Relative difference in ART coverage between MSM and all males. |  | 0.3-0.75 | | In the Mpumalanga Men’s study (2012-2013), 13.6% (95%CI: 7.8-21.5) and 9.7% (95% CI: 3.6-19.9) of MSM testing HIV positive in Gert Sibande and Ehlanzeni, respectively, self-reported currently taking ART (Lane, Osmand et al. 2014). In the Sibanye Health Project (2015), 11.4% (95%CI: 6.6-19.1) and 27.8% (95% CI: 22.7-33.5) of MSM testing HIV positive in Cape Town and Port Elisabeth, respectively, self-reported currently taking ART. In the Sibanye Health Project (2015), 22.9% (95%CI: 15.8-31.9) and 22.1% (95% CI: 17.4-27.6) of HIV +ve MSM were virally supressed in Cape Town and Port Elisabeth, respectively. In HPTN 075 (2015/16), ARV drugs were detected in 27.4% (95%CI: 18.2-38.2) and 25.0% (95%CI: 13.2-40.3) HIV positive MSM in Soweto and Cape Town, respectively (Zhang, Fogel et al. 2018).  UNAIDS give an ART coverage among men of 32% (23-40) in 2012 and 43% (32-54) in 2015; so we assume MSM have ART coverage of 0.3-0.75 that of all males. |
| Proportion of MSM that are circumcised. | $\zeta$ | Time varying | Not applicable. | See section on circumcision trends. |

## Supplementary Table 5. HIV epidemiological parameters. FSW denoted female sex workers; MSM denotes men who have sex with men.

| Parameter | Range | Reference |
| --- | --- | --- |
| HIV transmission probability per receptive vaginal sex act | 0.0006-0.006 | (Boily, Baggaley et al. 2009, Hughes, Baeten et al. 2012) |
| HIV transmission probability per insertive vaginal sex act from female to male | 0.0006-0.006 | (Boily, Baggaley et al. 2009, Hughes, Baeten et al. 2012) |
| Relative risk of acquiring HIV from receptive anal intercourse I vs. receptive vaginal intercourse | 2.0-18.0 | (Boily, Baggaley et al. 2009, Baggaley, White et al. 2010) |
| Relative risk of acquiring HIV from insertive anal intercourse vs. insertive vaginal intercourse | 1.0-2.0 | (Boily, Baggaley et al. 2009, Baggaley, White et al. 2010) |
| Relative infectiousness of those in the acute stage of HIV infection compared with the chronic stage | 4.45-18.8 | (Boily, Baggaley et al. 2009) |
| Relative infectiousness of those in the pre-AIDS and AIDS stages of HIV infection compared with the chronic stage | 4.45-11.88 | (Boily, Baggaley et al. 2009) |
| Efficacy of ART in reducing HIV transmission risk. | 0.70-1.0 | From a summary of studies that reported proportion virally suppressed on ART in South Africa. (Barth, Tempelman et al. 2011, Barnabas, van Rooyen et al. 2014, Fatti, Mothibi et al. 2014, Shearer, Brennan et al. 2014, Abuelezam, McCormick et al. 2016, Jean, Puren et al. 2016, Johnson, Chiu et al. 2016, Kapiamba, Masango et al. 2016, Lecher 2016, Lippman, Shade et al. 2016, Moyo, Chasela et al. 2016, Coetzee, Hunt et al. 2017, Huerga, Shiferie et al. 2017, Tsondai, Wilkinson et al. 2017, Zanoni, Sibaya et al. 2017) |
| Duration of acute stage in months | 1.2-6 | (Hollingsworth, Anderson et al. 2008) |
| Duration of pre-AIDS stage in months when not on ART | 4.8-14.0 | (Hollingsworth, Anderson et al. 2008) |
| Duration of AIDS stage in months when not on ART | 6.9-12.7 | (Hollingsworth, Anderson et al. 2008) |
| ART recruitment rate | Varied | Varied to fit ART coverage in the different age groups. |
| Median time to death when not on ART per year | 8.7-14.2 | (Todd, Glynn et al. 2007) |
| Factor difference in HIV related death when on ART compared to off ART | 0.1-0.45 | Systematic search showed that HIV patients with CD4 counts 200-350 can expect to live 4.8 life years without treatment but have an expected net benefit of 14.5 life years on treatment (Johansson, Robberstad et al. 2010)  Crude mortality rate for individuals who started cART and followed up for a median of 48 months and 24 months for different years of enrolled was 31.8 (95%CI: 30.3-33.2) per 1000 pyrs (Mills, Bakanda et al. 2011). |
| Loss to follow up on ART for general population per year | 6.4-18.2% | Of those who initiated ART in < 90 days 8% (15/190) in the standard arm group were lost to follow up at 10-month visit (Rosen, Maskew et al. 2016). Therefore, the loss to follow up from those in the standard arm was 11.3%(6.4-18.2%) per year.  In a clinic in Gauteng Province, South Africa, a retrospective observational cohort showed incidence of loss to follow up of 109 per 1000 person-years (95%CI: 92-128) in general population (Mberi, Kuonza et al. 2015). We take the lower and upper bound from the two studies. |
| Loss to follow up on ART for MSM per year | Assume the same LTFU rate for MSM as the general population | A study in Johannesburg found there were no statistically significant differences in ART retention between MSM and other men. (Rees, Radebe et al. 2017) |
| Loss to follow up on ART for FSW per year | 30.2% (95%CI: 22.7-38.6%) | The TAPs study among FSW showed that loss to follow up on early ART was 30.2% (22.7-38.6%) in the first 12 months out of 139 FSW enrolled onto ART (Eakle, Gomez et al. 2017) |
| Efficacy (%) of circumcision for reducing HIV susceptibility among men | 54% (95%CI: 38-66%) | (Siegfried, Muller et al. 2009)  For sex between men, we assume that male circumcision reduces HIV acquisition risk during insertive anal sex only. Based on limited data of the efficacy of circumcision on HIV acquisition risk among MSM by their preferred sexual positioning(Templeton, Jin et al. 2009, Sánchez, y Rosas et al. 2011) , we assume the same efficacy for MSM during insertive anal sex as heterosexual sex. |
| Per-sex-act (%) efficacy of condoms in reducing HIV transmission risk in vaginal and anal sex | 80% (95%CI: 66-94%) | (Weller and Davis 2001) |
| Non-HIV death rate – depending on gender. | Varied -time dependant | (United Nation, Department of Economic and Social Affairs et al. 2015) |
