# Supplementary material for: Estimating the contribution of key populations towards HIV transmission in South Africa
Source: J Int AIDS Soc. 2021 Feb 2;24(1):e25650. doi: 10.1002/jia2.25650 (PMC7855076; doi:10.1002/jia2.25650)
Supplement: Supplementary file 2 — Figure S1. a, Modelled condom use trends for low risk males and females. Continuous black line indicates median projections from all the baseline model fits with pink shaded areas showing 95% credibility intervals. Vertical black lines show the prior ranges. b, Modelled condom use trends for female sex workers (FSW). Both figures show condom use for vaginal intercourse (VI). FSW condom use with casual partners for VI is 1.25 to 1.75 times that for main partners VI; condom use for AI with main/casual partners is assumed to be the same as VI; condom use for AI with commercial partners is assumed to be 0.5 to 1.0 times that of commercial VI. Continuous black line indicates median projections from all the baseline model fits with pink shaded areas showing 95% credibility intervals. Vertical black lines show the prior ranges. c, Modelled condom use trends for men how have sex with men (MSM) with their male and female regular and casual partners. Continuous black line indicates median projections from all the baseline model fits with pink shaded areas showing 95% credibility intervals. Vertical black lines show the prior ranges. d, Modelled condom use trends for Clients with their main and casual partners. Condom use is assumed to be the same for vaginal intercourse and anal intercourse. Continuous black line indicates median projections from all the baseline model fits with pink shaded areas showing 95% credibility intervals. Vertical black lines show the prior ranges. Figure S2. Modelled ART trend for female sex workers (FSW), men who have sex with men (MSM) and low risk females and males. Continuous black line indicates median projections from all the baseline model fits with pink shaded areas showing 95% credibility intervals. Vertical black lines show UNAIDS estimates Figure S3. A comparison of model fits with HIV prevalence estimates for (a) younger men who have sex with men (MSM), and (b) older MSM. Continuous black line shows median projections from all the model [file JIA2-24-e25650-s002.docx]

**Supplementary Figures to: Estimating the contribution of key populations towards HIV transmission in South Africa**


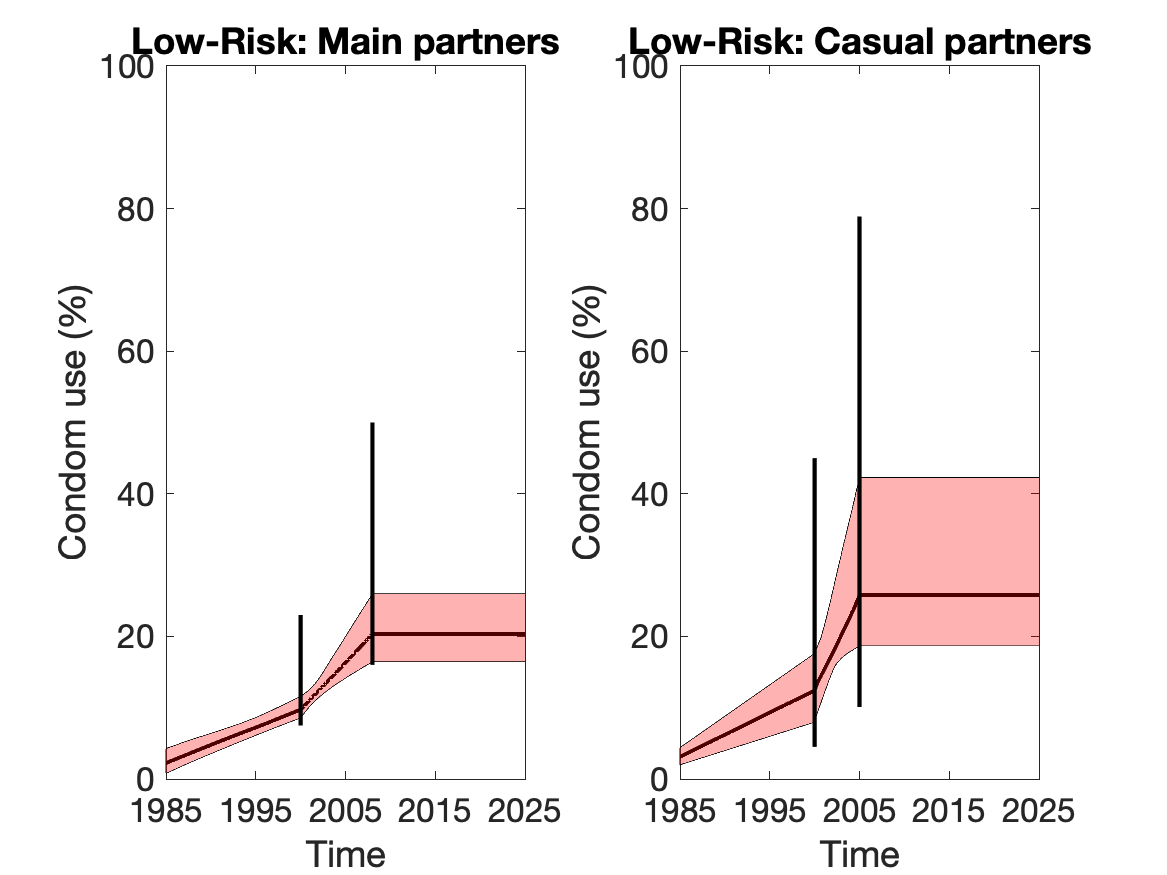


Supplementary Figure 1a: Modelled condom use trends for low risk males and females. Continuous black line indicates median projections from all the baseline model fits with pink shaded areas showing 95% credibility intervals. Vertical black lines show the prior ranges.


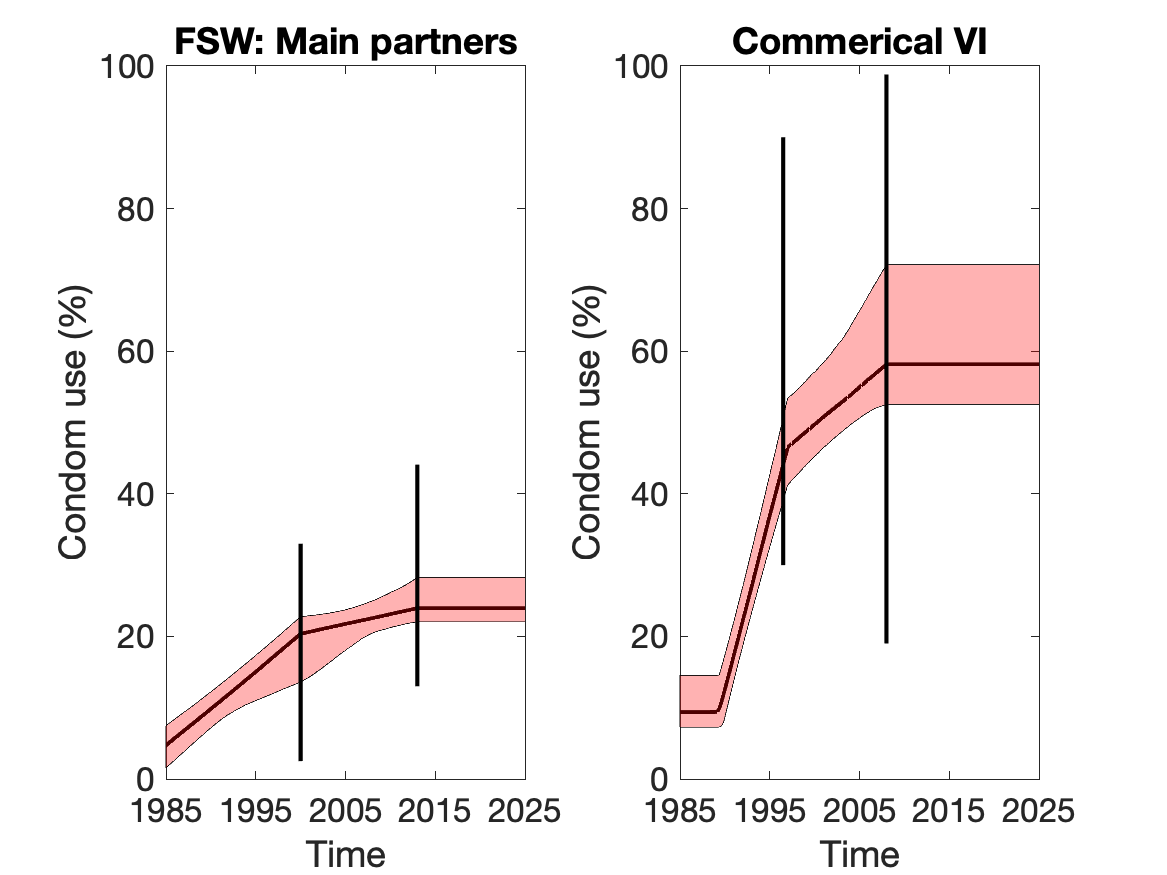


Supplementary Figure 1b. Modelled condom use trends for female sex workers (FSW). Both figures show condom use for vaginal intercourse (VI). FSW condom use with casual partners for VI is 1.25-1.75 times that for main partners VI; condom use for AI with main/casual partners is assumed to be the same as VI; condom use for AI with commercial partners is assumed to be 0.5-1.0 times that of commercial VI. Continuous black line indicates median projections from all the baseline model fits with pink shaded areas showing 95% credibility intervals. Vertical black lines show the prior ranges.


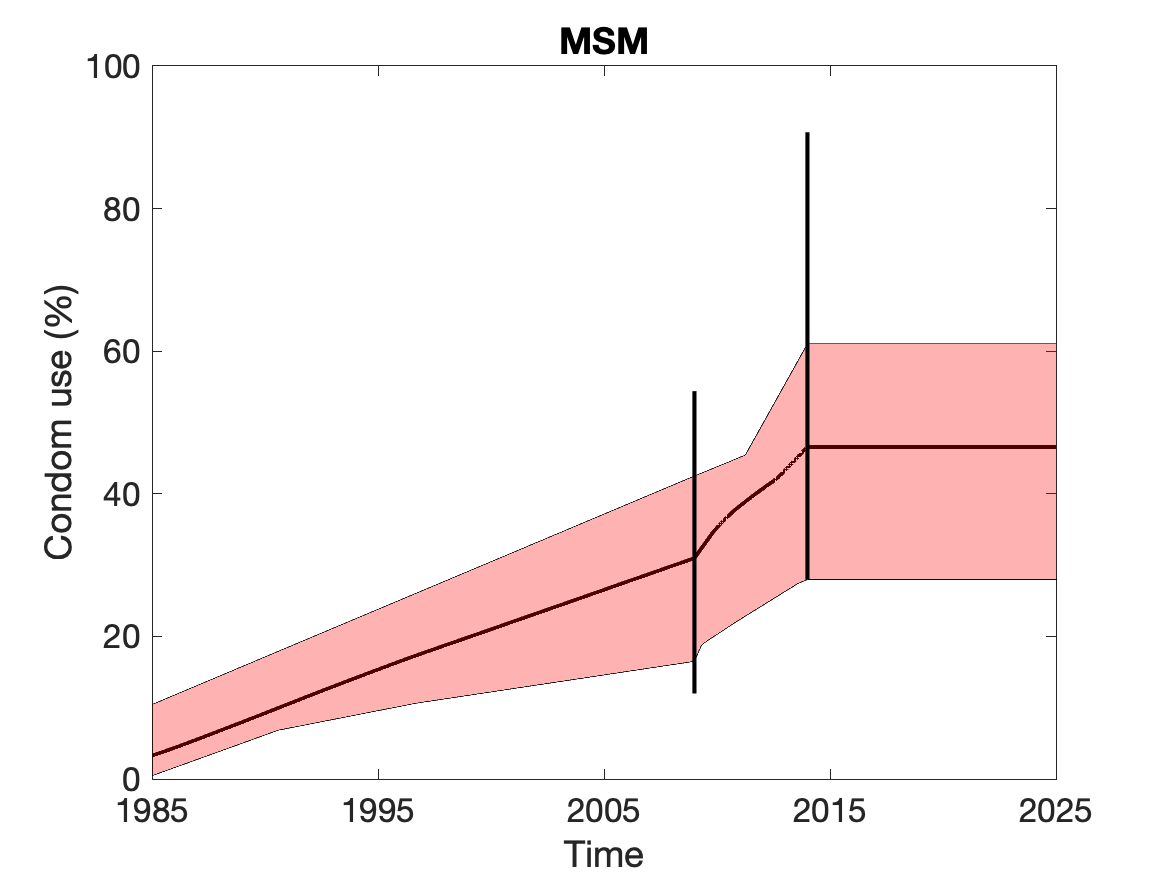


Supplementary Figure 1c. Modelled condom use trends for men how have sex with men (MSM) with their male and female regular and casual partners. Continuous black line indicates median projections from all the baseline model fits with pink shaded areas showing 95% credibility intervals. Vertical black lines show the prior ranges.


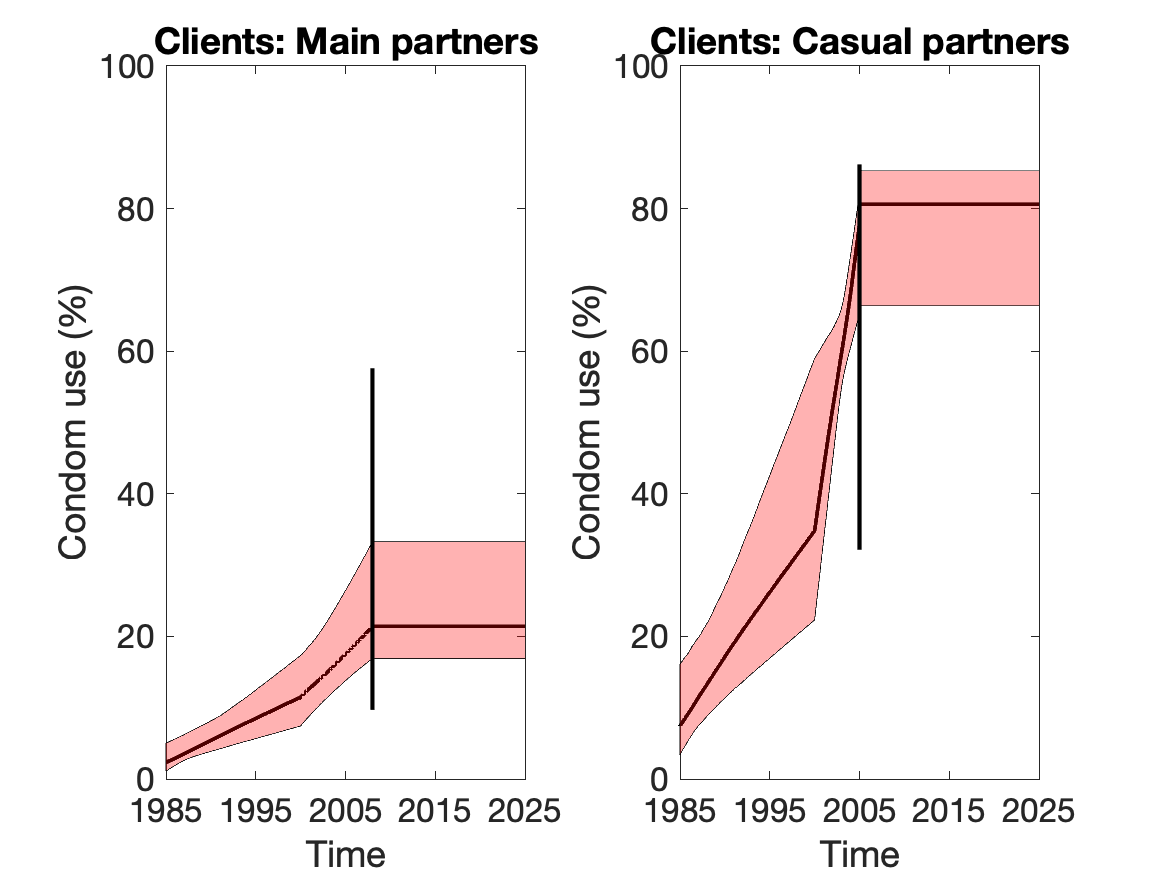


Supplementary Figure 1d. Modelled condom use trends for Clients with their main and casual partners. Condom use is assumed to be the same for vaginal intercourse and anal intercourse. Continuous black line indicates median projections from all the baseline model fits with pink shaded areas showing 95% credibility intervals. Vertical black lines show the prior ranges.


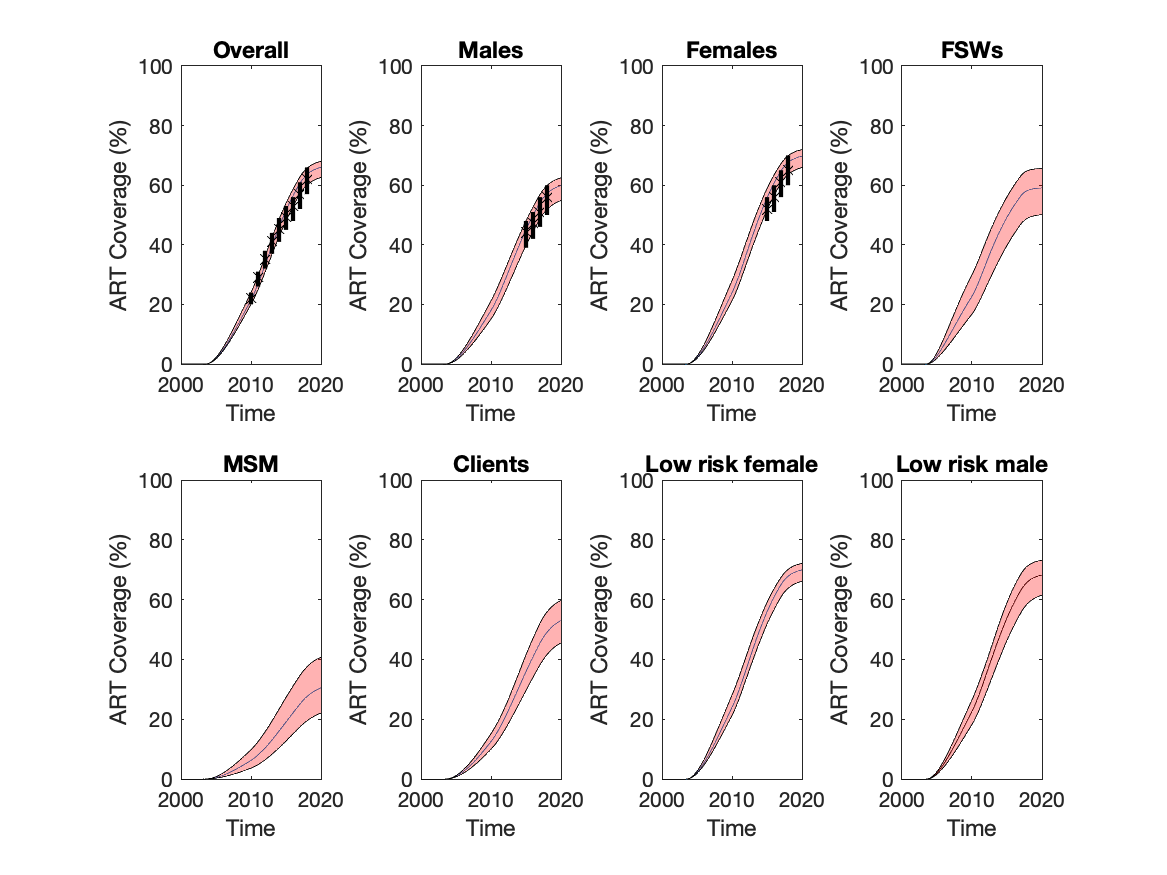


Supplementary Figure 2: Modelled ART trend for female sex workers (FSW), men who have sex with men (MSM) and low risk females and males. Continuous black line indicates median projections from all the baseline model fits with pink shaded areas showing 95% credibility intervals. Vertical black lines show UNAIDS estimates.

Supplementary Figure 3: A comparison of model fits with HIV prevalence estimates for (a) younger men who have sex with men (MSM), and (b) older MSM. Continuous black line shows median projections from all the model fits, with the grey shaded areas showing 95% credibility intervals. Red points and whiskers show regional HIV prevalence data points with 95% confidence intervals. Most available MSM data is heavily weighted towards young MSM (e.g. In South African RDS studies of MSM, the median proportion of MSM who are aged <25 is 70%) and so it is to be expected that older MSM will not fit the data so well.


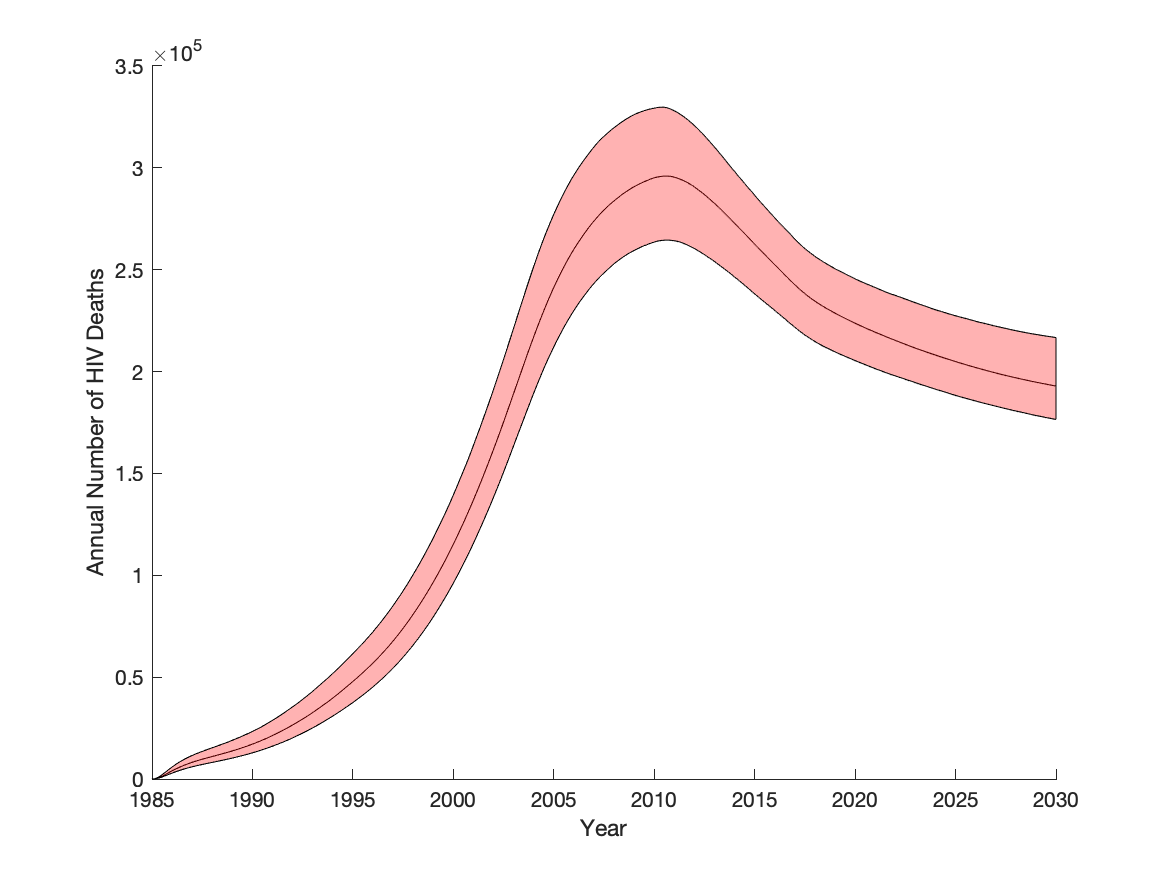


Supplementary Figure 4: Model projections of the number of annual HIV deaths. Continuous black line indicates median projections from all the baseline model fits with pink shaded areas showing 95% credibility intervals.


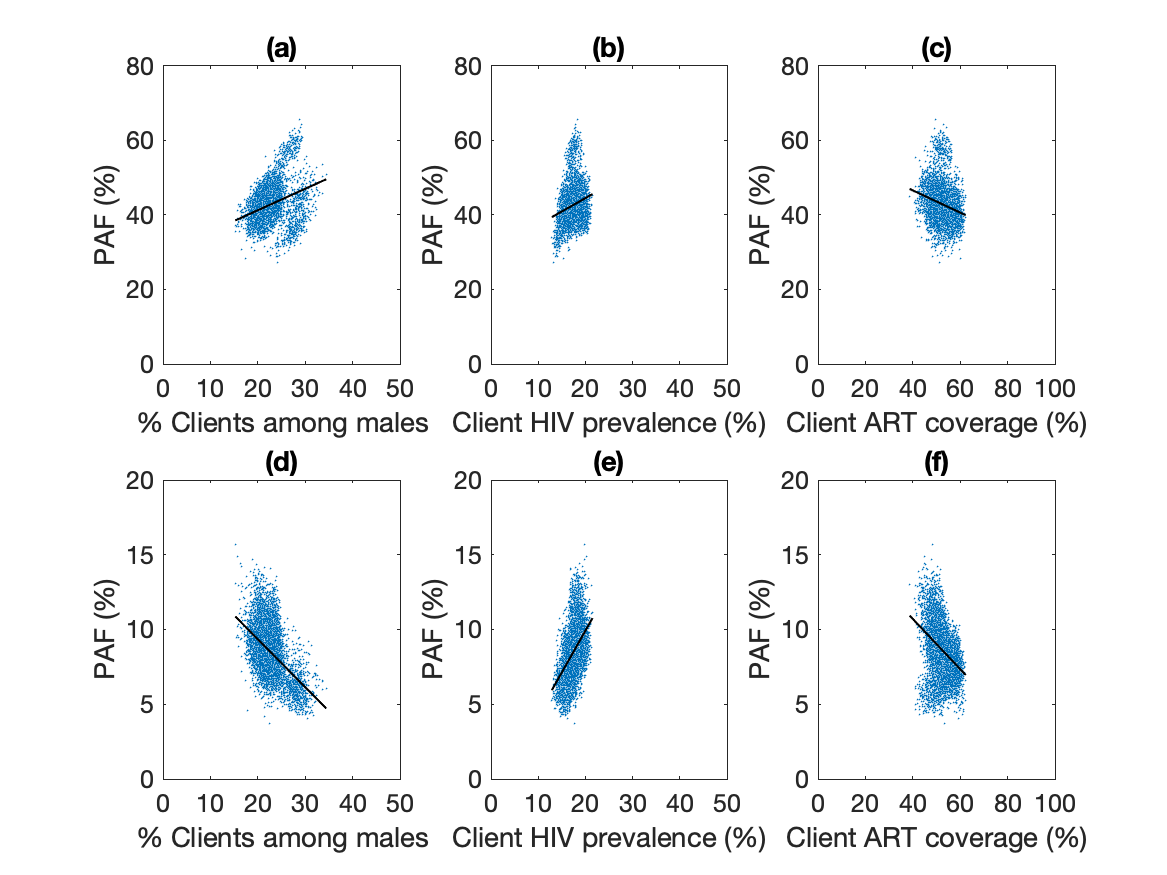


Supplementary Figure 5: Scatter plots showing the association between: (a) client population size in 2020 and the population attributable fraction (PAF) of non-commercial sex among clients from 2020-2029; (b) HIV prevalence among clients in 2020 and the PAF of non-commercial sex among clients from 2020-2029; (c) ART coverage among clients in 2020 and the PAF of non-commercial sex among clients from 2020-2029; (d) client population size in 2020 and the PAF of commercial sex from 2020-2029; (e) HIV prevalence among clients in 2020 and the PAF of commercial sex from 2020-2029; (f) ART coverage among clients in 2020 and the PAF of commercial sex from 2020-2029. Black lines are the least-squares line.

Supplementary Figure 6: ANCOVA Results: Contribution of uncertainty in each parameter to the variability in the PAF of non-commercial sex among clients over 2010-2019. Parameters accounting for less than 1% of the uncertainty are not shown. MSM denotes men who have sex with men.

Supplementary Figure 7: ANCOVA Results: Contribution of uncertainty in each parameter to the variability in the PAF of commercial sex over 2010-2019. Parameters accounting for less than 1% of the uncertainty are not shown. FSW denotes female sex workers; MSM denotes men who have sex with men; LRM denotes low risk males.

Supplementary Figure 8: ANCOVA Results: Contribution of uncertainty in each parameter to the variability in the PAF of sex between low-risk groups over 2010-2019. Parameters accounting for less than 1% of the uncertainty are not shown. MSM denotes men who have sex with men.
